# A DMRG/CASPT2 Investigation of Metallocorroles: Quantifying Ligand Noninnocence in Archetypal 3d and 4d Element Derivatives

**DOI:** 10.1021/jacsau.1c00417

**Published:** 2021-10-21

**Authors:** Quan Manh Phung, Yasin Muchammad, Takeshi Yanai, Abhik Ghosh

**Affiliations:** † Department of Chemistry, Graduate School of Science, Nagoya University, Furo-cho, Chikusa-ku, Nagoya, Aichi 464-8602, Japan; ‡ Institute of Transformative Bio-Molecules (WPI-ITbM), Nagoya University, Furo-cho, Chikusa-ku, Nagoya, Aichi 464-8602, Japan; § Department of Chemistry, UiT-The Arctic University of Norway, N-9037 Tromsø, Norway

**Keywords:** DMRG, CASSCF, CASPT2, corrole, metallocorrole, nitrosyl, ligand noninnocence, noninnocent ligand

## Abstract

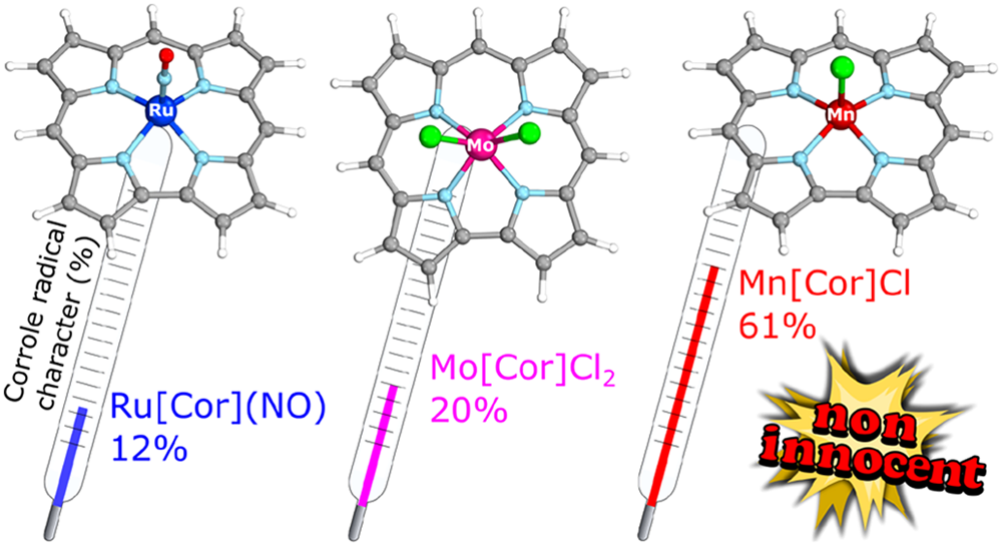

Hybrid density functional theory (B3LYP) and density matrix renormalization group (DMRG) theory have been used to quantitatively compare the degree of ligand noninnocence (corrole radical character) in seven archetypal metallocorroles. The seven complexes, in decreasing order of corrole noninnocent character, are Mn[Cor]Cl > Fe[Cor]Cl > Fe[Cor](NO) > Mo[Cor]Cl_2_ > Ru[Cor](NO) ≈ Mn[Cor]Ph ≈ Fe[Cor]Ph ≈ 0, where [Cor] refers to the unsubstituted corrolato ligand. DMRG-based second-order perturbation theory calculations have also yielded detailed excited-state energetics data on the compounds, shedding light on periodic trends involving middle transition elements. Thus, whereas the ground state of Fe[Cor](NO) (*S* = 0) is best described as a locally *S* = 1/2 {FeNO}^7^ unit antiferromagnetically coupled to a corrole A′ radical, the calculations confirm that Ru[Cor](NO) may be described as simply {RuNO}^6^–Cor^3–^, that is, having an innocent corrole macrocycle. Furthermore, whereas the ferromagnetically coupled *S* = 1{FeNO}^7^–Cor^•2–^ state of Fe[Cor](NO) is only ∼17.5 kcal/mol higher than the *S* = 0 ground state, the analogous triplet state of Ru[Cor](NO) is higher by a far larger margin (37.4 kcal/mol) relative to the ground state. In the same vein, Mo[Cor]Cl_2_ exhibits an adiabatic doublet-quartet gap of 36.1 kcal/mol. The large energy gaps associated with metal–ligand spin coupling in Ru[Cor](NO) and Mo[Cor]Cl_2_ reflect the much greater covalent character of 4d−π interactions relative to analogous interactions involving 3d orbitals. As far as excited-state energetics is concerned, DMRG-CASPT2 calculations provide moderate validation for hybrid density functional theory (B3LYP) for qualitative purposes, but underscore the possibility of large errors (>10 kcal/mol) in interstate energy differences.

## Introduction

Over a half century ago, the Danish chemist C. K. Jørgensen defined noninnocent ligands (although he called them “suspect”) as those that leave the oxidation state of the coordinated atom uncertain or debatable.^1^ The resulting complexes defy description in terms of a single Lewis structure. Esoteric as it initially sounded, noninnocent ligands promptly entered the mainstream of coordination chemistry^2^ and today are utterly ubiquitous.^3^ Aside from its intrinsic theoretical interest, chemists’ growing appreciation of the phenomenon has also led to impressive applications in catalysis.^4,5^ Unfortunately, the subtlety of the phenomenon has often led to its being overlooked or mischaracterized.^6^ The need for more sharply defined terminology has also become apparent: little is gained, for example, by designating full-fledged radicals^7^ (exemplified, perhaps most famously, by the Cu^II^-phenoxy-radical active form of galactose oxidase^8,9^), even if bound to a metal, as noninnocent. The more general term metalloradical may be appropriate in such cases. On the contrary, a great deal of insight may be obtained by rigorously quantifying noninnocence. Doing so would allow us to rank metal complexes in terms of ligand noninnocence, either for a given ligand as a function of different coordinated metals or for a given metal as a function of systematic variations to the ligand’s structure. Herein we describe the realization of this objective for a series of unsubstituted metallocorroles, and, as hoped for, the analysis proved productive, immediately affording multiple conceptual spin-offs (as described below).

**Scheme 1:**
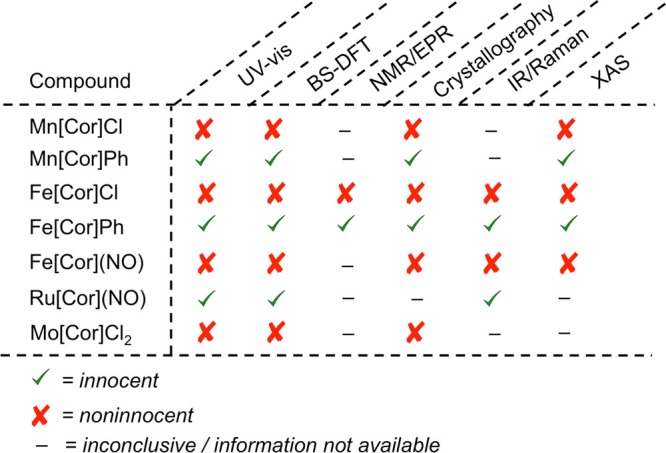
Summary of Existing Evidence Pertaining to Ligand Noninnocence Relevant to Complexes Studied in This Work

Metallocorroles,^10−12^ in recent years, have provided several paradigmatic examples of noninnocent ligands, in which the macrocycle is best described as having partial corrole^•2–^ character. The systems in question, MnCl,^13,14^ FeCl^13,15−19^ FeNO,^20,21^ Fe_2_(μ-O),^22^ Co-py^23^/DMSO^24^ (py = pyridine), Co-PPh_3_,^25^ and Cu^26−34^ corroles, largely involve 3d transition metals,^35^ although certain 4d/5d element complexes (e.g., Ag^36,37^ and MoCl_2_^38,39^ corroles) are also thought to involve noninnocent corroles. In one of our laboratories, we have long used a suite of experimental probes, including UV–vis, nuclear magnetic resonance/electron paramagnetic resonance (NMR/EPR), infrared (IR)/Raman, and X-ray absorption (XANES) spectroscopies, and single-crystal X-ray structures to diagnose noninnocent character with a high degree of certitude (Scheme 1).^6^ Broken-symmetry (BS) density functional theory (DFT) calculations have nicely complemented the experimental measurements, helping visualize spin density distributions, where relevant.^6^ For FeCl,^40^ FeNO,^41^ and Cu^42^ corroles, CASSCF/CASPT2 calculations have also been reported, illuminating the excited-state architecture of the compounds. It is worth noting that the CASSCF method has also been applied to a number of other noninnocent systems.^43−56^ Electrochemistry and UV–vis/IR spectroelectrochemistry have also afforded invaluable insight into the question of the innocent/noninnocent character of metallocorroles.^36,57,58^

**Scheme 2:**
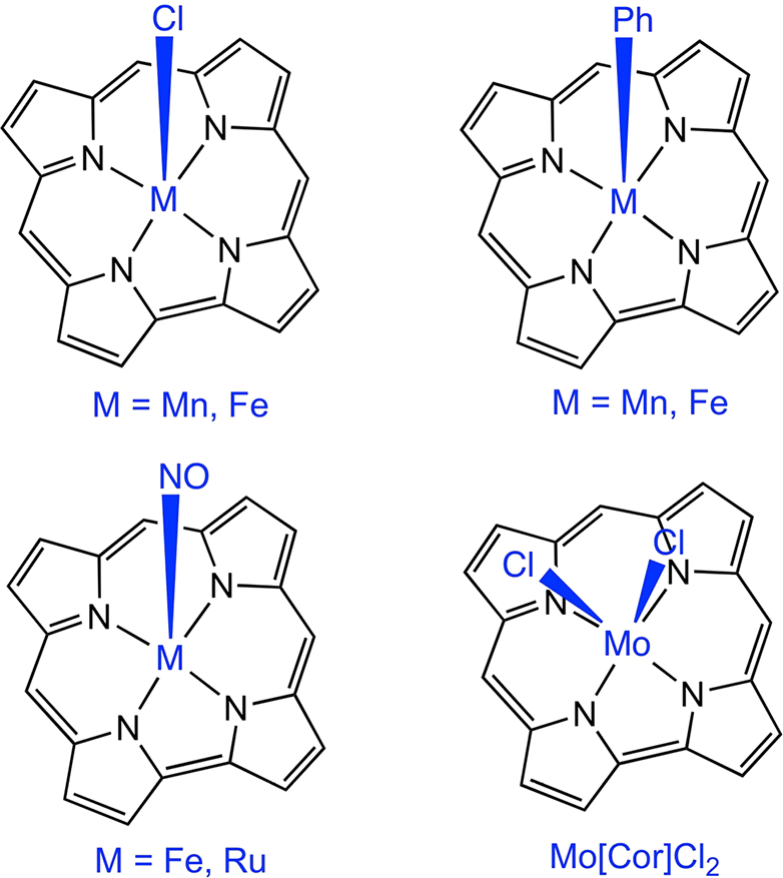
Molecules Studied in This Work

As alluded to above, a key lacuna remains in our electronic–structural appreciation of corroles in that we do not have a comparative picture of the degree of noninnocence of different metallocorrole systems. We have addressed this knowledge gap here with a combined DFT (B3LYP) and density matrix renormalization group (DMRG) study of seven archetypal middle transition metal corroles that may be reasonably expected to exhibit some degree of noninnocent character. These include: Mn[Cor]Cl and Mn[Cor]Ph; Fe[Cor]Cl, Fe[Cor]Ph, Fe[Cor](NO), and Ru[Cor](NO);^59^ and Mo[Cor]Cl_2_ (Scheme 2). Excluded from this study are the coinage metal corroles, where the qualitative picture also critically depends on peripheral substituents.^6,34,36^ A separate study is planned for these systems and will be communicated in due course. The results, for the first time, provide a comparative account of both the excited-state architecture (spin-state energetics) and ground-state noninnocence of the complexes. In so doing, the analysis affords some immediate conceptual spin-offs, most notably some of the first insights into metal–ligand spin coupling strengths in noninnocent 3d versus 4d transition metal complexes.

## Computational Methods

All structures were optimized with the B3LYP functional^60−65^ and D3 dispersion corrections with Becke–Johnson damping,^66,67^ as implemented in Turbomole v.7.4.^68,69^ The symmetry was generally exploited to reduce the computational cost. As a result, some optimized structures proved unstable; that is, they turned out to be transition states. (See the Supporting Information.) These were reoptimized without symmetry so that all final ground-state structures correspond to minima on their respective potential energy surfaces. For the optimizations, we used def2-TZVP basis sets for the metals and def2-SVP basis sets for all ligand atoms.^70^ Single-point B3LYP calculations were then performed with def2-TZVP basis sets for all atoms. Calculations with B3LYP* (a modified functional from B3LYP with only 15% of Hartree–Fock exchange proposed by Reiher et al.^71,72^) were also performed. We found that B3LYP* geometries are close to those obtained with B3LYP (Table S17), in agreement with the findings of Reiher et al.^71,72^

The optimized B3LYP structures were used as input in single-point DMRG-CASSCF/CASPT2^73−81^ calculations, which were performed with the OpenMolcas^82,83^ program system interfaced with the CheMPS2 library.^84^ We used the same computational settings as described in our previous work on Fe[Cor](NO).^41^ ANO-RCC basis sets^85,86^ contracted to [7s6p5d3f2g1h] for all metals; [4s3p2d1f] for C, N, and O; [5s4p2d1f] for Cl; and [3s1p] for H were employed. The Cholesky decomposition of two-electron integrals was used, with a threshold of 10^–6^ au.^87^ Scalar relativistic effects were taken into account with a second-order Douglas–Kroll–Hess (DKH) Hamiltonian.^88−90^ All DMRG calculations were performed with the default settings implemented in the OpenMolcas-CheMPS2 interface: Fiedler orbital ordering,^91^ a residual norm threshold of 10^–4^ for the Davidson algorithm, and perturbative noise with a prefactor of 0.05.^84^ The number of renormalized states *m* was 4000 in all calculations. Although this value was chosen based on our computational resources, it is expected to be sufficient for quantitatively accurate results.^41,92^ For example, in Fe[Cor](NO), the DMRG-CASPT2 triplet-singlet splitting fully converges to within 0.1 kcal/mol, even at *m* = 2000. In CASPT2 calculations, all core and semicore electrons (through 3s and 3p for Mn and Fe and through 4s and 4p for Ru and Mo) were kept frozen. A standard ionization potential electron affinity (IPEA) shift^93^ of 0.25 au and an imaginary shift^94^ of 0.1 au were used. The contribution of the semicore electrons to the correlation energy was calculated with UB3LYP-CCSD(T), that is, CCSD(T) on top of a UB3LYP reference, using the Orca v.4.2 program package.^95^ This combination of CASPT2 and CCSD(T) is similar to a recently proposed method CASPT2/CC.^96^ The advantage is that this spin-unrestricted variation of CASPT2/CC can be used to describe systems with antiferromagnetic coupling to some extent. Its performance for antiferromagnetic coupled Fe[Cor](NO) has been carefully evaluated.^41^ We finally note that in all complexes, the contributions of the semicore electron correlation to the relative energies are minimal (∼1 kcal/mol).

The active spaces were chosen based on well-established procedures^97^ and our previous studies on metalloporphyin^98^ and metallocorrole^41^ derivatives. Ideally, the active spaces for different states should be identical, consisting of all metal *n*d orbitals, all correlating (*n* + 1)d orbitals to account for the so-called double-shell effect,^97,99,100^ and all ligand orbitals that can interact with the metal *n*d orbitals. However, in a state with an empty metal *n*d orbital, the double-shell effect is so minor that the correlating (*n* + 1)d orbital in question tends to rotate out of the active space. In such cases, we removed the problematic (*n* + 1)d orbital from the active space. For Fe[Cor]Cl, the active space consists of 24 electrons in either 25 or 26 orbitals: five Fe(3d) orbitals, a maximum of five Fe(4d) orbitals, one σ(Fe–N) orbital, and all corrole π orbitals except for eight that are localized on corrole β-carbons. For Mn[Cor]Cl, the active space is either CAS(23,25) or CAS(23,24). For Fe[Cor]Ph and Mn[Cor]Ph, we extended the active space by including a σ(M–C) orbital describing the covalent interaction between the metal atom and the phenyl ring. In Ru[Cor](NO), the double-shell effect is negligible, as commonly seen in second-row transition metal complexes.^100,101^ Accordingly, we excluded the 5d orbitals from the active space of Ru[Cor](NO). A consequence of this exclusion is that the natural orbital occupation number (NOON) of the nonbonding Ru(4d_*xy*_) orbital is almost exactly two. We accordingly chose to keep this orbital inactive. The active space also contains 22 ligand orbitals: 15 corrole π orbitals, one σ(Ru–N) orbital, two NO(π) and two corresponding NO(π*) orbitals that can mix with Ru(4d) orbitals, and the NO(σ, σ*) pair. The final active space for Ru[Cor](NO) is CAS(30,26). In Mo[Cor]Cl_2_, the active space is CAS(25,23), including 15 corrole π, five Mo 4d, and three σ(Mo–N) orbitals. For the sake of completeness, we also carried out calculations on Fe[Cor](NO). The results are essentially identical to those obtained previously,^41^ with very minor differences resulting from slightly different optimized geometries, given that here we have employed the B3LYP functional incorporating the VWN(III) correlation functional. The active orbitals are depicted in Figures S3–S9. The formal occupation of each state is shown in Table 1. (Note that Table 1 uses the Enemark–Feltham notation for the two NO complexes,^102^ in which the superscript refers to the total number of metal d and NO π* electrons.)

**Table 1:** States Studied in This Work

state	formal occupation	metal and ligand spin states
Fe[Cor]Cl		
^7^A′	(3d_*xy*_)^↑^ (3d_*yz*_)^↑^ (3d_*xz*_)^↑^ (3d_*z*^2^_)^↑^ (3d_*x*^2^__–*y*^2^_)^↑^ (*a*′)^↑^	*S* = 5*/*2 Fe(III) F-coupled to A′ Cor^•2–^
^7^A″	(3d_*xy*_)^↑^ (3d_*yz*_)^↑^ (3d_*xz*_)^↑^ (3d_*z*^2^_)^↑^ (3d_*x*^2^__–*y*^2^_)^↑^ (*a*″)^↑^	*S* = 5*/*2 Fe(III) F-coupled to A″ Cor^•2–^
^5^A′	(3d_*xy*_)^↑^ (3d_*yz*_)^↑^ (3d_*xz*_)^↑^ (3d_*z*^2^_)^↑^ (3d_*x*^2^__–*y*^2^_)^↑^ (*a*′)^↓^	*S* = 5*/*2 Fe(III) AF-coupled to A′ Cor^•2–^
^5^A″	(3d_*xy*_)^2^ (3d_*yz*_)^↑^ (3d_*xz*_)^↑^ (3d_*z*^2^_)^↑^ (3d_*x*^2^__–*y*^2^_)^0^ (*a*′)^↑^	*S* = 3*/*2 Fe(III) F-coupled to A′ Cor^•2–^
^3^A′	(3d_*xy*_)^2^ (3d_*yz*_)^↑^ (3d_*xz*_)^↑^ (3d_*z*^2^_)^↑^ (3d_*x*^2^__–*y*^2^_)^0^ (*a*″)^↓^	*S* = 3*/*2 Fe(III) AF-coupled to A″ Cor^•2–^
^3^A″	(3d_*xy*_)^2^ (3d_*yz*_)^↑^ (3d_*xz*_)^↑^ (3d_*z*^2^_)^↑^ (3d_*x*^2^__–*y*^2^_)^0^ (*a*′)^↓^	*S* = 3*/*2 Fe(III) AF-coupled to A′ Cor^•2–^
^1^A′	(3d_*xy*_)^2^ (3d_*yz*_)^2^ (3d_*xz*_)^↑^ (3d_*z*^2^_)^0^ (3d_*x*^2^__–*y*^2^_)^0^ (*a*″)^↓^	*S* = 1*/*2 Fe(III) AF-coupled to A″ Cor^•2–^
Mn[Cor]Cl		
^6^A′	(3d_*xy*_)^↑^ (3d_*yz*_)^↑^ (3d_*xz*_)^↑^ (3d_*z*^2^_)^↑^ (3d_*x*^2^__–*y*^2^_)^0^ (*a*″)^↑^	*S* = 2 Mn(III) F-coupled to A″ Cor^•2–^
^6^A″	(3d_*xy*_)^↑^ (3d_*yz*_)^↑^ (3d_*xz*_)^↑^ (3d_*z*^2^_)^↑^ (3d_*x*^2^__–*y*^2^_)^0^ (*a*′)^↑^	*S* = 2 Mn(III) F-coupled to A′ Cor^•2–^
^4^A′	(3d_*xy*_)^↑^ (3d_*yz*_)^↑^ (3d_*xz*_)^↑^ (3d_*z*^2^_)^↑^ (3d_*x*^2^__–*y*^2^_)^0^ (*a*″)^↓^	*S* = 2 Mn(III) AF-coupled to A″ Cor^•2–^
^4^A″	(3d_*xy*_)^↑^ (3d_*yz*_)^↑^ (3d_*xz*_)^↑^ (3d_*z*^2^_)^↑^ (3d_*x*^2^__–*y*^2^_)^0^ (*a*′)^↓^	*S* = 2 Mn(III) AF-coupled to A′ Cor^•2–^
^2^A′	(3d_*xy*_)^2^ (3d_*yz*_)^↑^ (3d_*xz*_)^↑^ (3d_*z*^2^_)^0^ (3d_*x*^2^__–*y*^2^_)^0^ (*a*″)^↓^	*S* = 1 Mn(III) AF-coupled to A″ Cor^•2–^
^2^A″	(3d_*xy*_)^2^ (3d_*yz*_)^↑^ (3d_*xz*_)^↑^ (3d_*z*^2^_)^0^ (3d_*x*^2^__–*y*^2^_)^0^ (*a*′)^↓^	*S* = 1 Mn(III) AF-coupled to A′ Cor^•2–^
Fe[Cor](NO)		
^3^A	(3d_*xy*_)^2^ (3d_*yz*_+NO(π*))^2^ (3d_*xz*_+NO(π*))^2^ (3d_*z*^2^_)^↑^ (Cor-π)^↑^	*S* = 1*/*2 {Fe(NO)}^7^ F-coupled to Cor^•2–^
^1^A	(3d_*xy*_)^2^ (3d_*yz*_+NO(π*))^2^ (3d_*xz*_+NO(π*))^2^ (3d_*z*^2^_)^↑^ (Cor-π)^↓^	*S* = 1*/*2 {Fe(NO)}^7^ AF-coupled to Cor^•2–^
Ru[Cor](NO)		
^3^A	(4d_*xy*_)^2^ (4d_*yz*_+NO(π*))^2^ (4d_*xz*_+NO(π*))^2^ (4d_*z*^2^_)^↑^ (Cor-π)^↑^	*S* = 1*/*2 {Ru(NO)}^7^ F-coupled to Cor^•2–^
^1^A′	(4d_*xy*_)^2^ (4d_*yz*_+NO(π*))^2^ (4d_*xz*_+NO(π*))^2^ (4d_*z*^2^_)^0^	*S* = 0 {Ru(NO)}^6^ Cor^3–^
Fe[Cor]Ph		
^7^A′	(3d_*xy*_)^↑^ (3d_*yz*_)^↑^ (3d_*xz*_)^↑^ (3d_*z*^2^_)^↑^ (3d_*x*^2^__–*y*^2^_)^↑^ (*a*′)^↑^	*S* = 5*/*2 Fe(III) F-coupled to A′ Cor^•2–^
^7^A″	(3d_*xy*_)^↑^ (3d_*yz*_)^↑^ (3d_*xz*_)^↑^ (3d_*z*^2^_)^↑^ (3d_*x*^2^__–*y*^2^_)^↑^ (*a*″)^↑^	*S* = 5*/*2 Fe(III) F-coupled to A″ Cor^•2–^
^5^A′	(3d_*xy*_)^↑^ (3d_*yz*_)^↑^ (3d_*xz*_)^↑^ (3d_*z*^2^_)^↑^ (3d_*x*^2^__–*y*^2^_)^↑^ (*a*′)^↓^	*S* = 5*/*2 Fe(III) AF-coupled to A′ Cor^•2–^
^5^A″	(3d_*xy*_)^2^ (3d_*yz*_)^↑^ (3d_*xz*_)^↑^ (3d_*z*^2^_)^↑^ (3d_*x*^2^__–*y*^2^_)^0^ (*a*′)^↑^	*S* = 3*/*2 Fe(III) F-coupled to A′ Cor^•2–^
^3^A′	(3d_*xy*_)^2^ (3d_*yz*_)^2^ (3d_*xz*_)^↑^ (3d_*z*^2^_)^0^ (3d_*x*^2^__–*y*^2^_)^0^ (*a*″)^↑^	*S* = 1*/*2 Fe(III) F-coupled to A″ Cor^•2–^
^3^A″	(3d_*xy*_)^2^ (3d_*yz*_)^↑^ (3d_*xz*_)^↑^ (3d_*z*^2^_)^0^ (3d_*x*^2^__–*y*^2^_)^0^	*S* = 1 Fe(IV) Cor^3–^
^1^A′	(3d_*xy*_)^2^ (3d_*yz*_)^2^ (3d_*xz*_)^↑^ (3d_*z*^2^_)^0^ (3d_*x*^2^__–*y*^2^_)^0^ (*a*″)^↓^	*S* = 1*/*2 Fe(III) AF-coupled to A″ Cor^•2–^
Mn[Cor]Ph		
^6^A′	(3d_*xy*_)^↑^ (3d_*yz*_)^↑^ (3d_*xz*_)^↑^ (3d_*z*^2^_)^↑^ (3d_*x*^2^__–*y*^2^_)^0^ (*a*″)^↑^	*S* = 2 Mn(III) F-coupled to A″ Cor^•2–^
^6^A″	(3d_*xy*_)^↑^ (3d_*yz*_)^↑^ (3d_*xz*_)^↑^ (3d_*z*^2^_)^↑^ (3d_*x*^2^__–*y*^2^_)^0^ (*a*′)^↑^	*S* = 2 Mn(III) F-coupled to A′ Cor^•2–^
^4^A′	(3d_*xy*_)^2^ (3d_*yz*_)^↑^ (3d_*xz*_)^↑^ (3d_*z*^2^_)^0^ (3d_*x*^2^__–*y*^2^_)^0^ (*a*″)^↑^	*S* = 1 Mn(III) F-coupled to A″ Cor^•2–^
^4^A″	(3d_*xy*_)^↑^ (3d_*yz*_)^↑^ (3d_*xz*_)^↑^ (3d_*z*^2^_)^0^ (3d_*x*^2^__–*y*^2^_)^0^	*S* = 3*/*2 Mn(IV) Cor^3–^
^2^A′	(3d_*xy*_)^2^ (3d_*yz*_)^↑^ (3d_*xz*_)^↑^ (3d_*z*^2^_)^0^ (3d_*x*^2^__–*y*^2^_)^0^ (*a*″)^↓^	*S* = 1 Mn(III) AF-coupled to A″ Cor^•2–^
^2^A″	(3d_*xy*_)^2^ (3d_*yz*_)^↑^ (3d_*xz*_)^↑^ (3d_*z*^2^_)^0^ (3d_*x*^2^__–*y*^2^_)^0^ (*a*′)^↓^	*S* = 1 Mn(III) AF-coupled to A′ Cor^•2–^
Mo[Cor]Cl_2_		
^4^A′	(4d_*xy*_)^↑^ (4d_*yz*_)^0^ (4d_*xz*_)^0^ (4d_*z*^2^_)^↑^ (4d_*x*^2^__–*y*^2^_)^0^ (*a*′)^↑^	*S* = 1 Mo(IV) F-coupled to A′ Cor^•2–^
^4^A″	(4d_*xy*_)^↑^ (4d_*yz*_)^0^ (4d_*xz*_)^0^ (4d_*z*^2^_)^↑^ (4d_*x*^2^__–*y*^2^_)^0^ (*a*″)^↑^	*S* = 1 Mo(IV) F-coupled to A″ Cor^•2–^
^2^A′	(4d_*xy*_)^↑^ (4d_*yz*_)^0^ (4d_*xz*_)^0^ (4d_*z*^2^_)^↑^ (4d_*x*^2^__–*y*^2^_)^0^ (*a*′)^↓^	*S* = 1 Mo(IV) AF-coupled to A′ Cor^•2–^
^2^A″	(4d_*xy*_)^2^ (4d_*yz*_)^0^ (4d_*xz*_)^0^ (4d_*z*^2^_)^0^ (4d_*x*^2^__–*y*^2^_)^0^ (*a*″)^↑^	*S* = 0 Mo(IV) A″ Cor^•2–^

The radical character of an orbital *i* was quantified via an equation proposed by Head-Gordon,^103^*f*_*i*_ = min(*n*_*i*_, 2 – *n*_*i*_) = 1 – |1 – *n*_*i*_|, where *n*_*i*_ is the NOON calculated with either DFT or DMRG-CASSCF theory. A singly occupied orbital has an *f* value of one (or 100%), and a strictly doubly occupied or empty orbital has a radical character of zero. To evaluate the radical character of corrole, we considered two cases. In the first case, a corrole(π) orbital is singly occupied, and this orbital does not mix appreciably with metal orbitals. Therefore, *f* is close to unity, and we consider the radical character of corrole as one. In states with a metal d orbital antiferromagnetically coupled to a corrole(π) orbital, strong mixing between the two orbitals is expected, giving rise to a bonding/antibonding pair of metal(d) ± corrole(π) orbitals. In such a case, the radical character of the corrole is estimated to be *half* of the total radical character of this pair of orbitals. This approach gives results identical to those obtained in other studies,^41,104,105^ which employed the equation *f* = 1 – 0.5(*n*^+^ – *n*^–^), where *n*^+^ and *n*^–^ are the NOONs of the bonding and antibonding MOs, respectively (*n*^+^ + *n*^–^ ≈ 2).

## Results and Discussion

### Excited-State Energetics

All B3LYP and DMRG-CASPT2/CC results are summarized in Table 2. For each state, we have shown the relative adiabatic energy Δ*E* with respect to the ground state (GS), the spin expectation value ⟨*S*^2^⟩, the natural spin populations of the metal center, corrole, and nitrosyl, and the radical character of the corrole macrocycle. As far as excited-state energetics is concerned, the overall agreement between B3LYP and DMRG-CASPT2/CC calculations may be described as fair, even though KS-DFT is known for its inconsistent performance vis-à-vis the spin state energetics of transition metal complexes.^106−111^ Thus B3LYP correctly predicts the GS for all complexes, relative to DMRG-CASPT2/CC results and to experimental evidence,^6^ namely, ^3^A″ for Fe[Cor]Cl and Fe[Cor]Ph, ^4^A″ for Mn[Cor]Cl and Mn[Cor]Ph, ^1^A for Fe[Cor](NO), ^1^A′ for Ru[Cor](NO), and ^2^A′ for Mo[Cor]Cl_2_. This result, however, is not particularly surprising, considering that these complexes do not have exceptionally low-lying excited states. The lowest DMRG-CASPT2/CC gaps between the GS and the first excited state (Δ*E*_1_) are found in Fe[Cor]Cl and Mn[Cor]Cl, being 4.4 and 9.2 kcal/mol, respectively. In Fe[Cor]Ph and Mn[Cor]Ph, Δ*E*_1_ increases to 18.7 and 38.7 kcal/mol, respectively, reflecting the stronger covalent interaction between the metal atom and the axial ligand. In Fe[Cor](NO), the singlet–triplet gap is 17.5 kcal/mol,^41^ whereas in Ru[Cor](NO) and Mo[Cor]Cl_2_, the energy gap between the antiferromagnetically and ferromagnetically coupled states increases to ∼36 kcal/mol. The larger gaps for the latter two complexes are related to greater covalence between 4d transition metals and their ligands and are indeed a reflection of the well-known tendency of second and third-row transition complexes of favoring low-spin (LS) over high-spin (HS) states.^112^ The value of the present insight derives substantially from its rarity: Detailed studies of metal–ligand covalence, especially ligand noninnocence, involving 4d/5d elements are exceptionally rare, so little quantitative information is available on the strength of metal–ligand spin couplings.^39,112^

**Table 2:** Properties of Various States Calculated with B3LYP and DMRG-CASPT2/CC: Relative Energies (in kcal/mol) with Respect to the Ground State, Spin Expectation Value, Natural Spin Populations, and the Radical Character of the Corrole Ring

	B3LYP						DMRG-CASPT2/CC	
			spin population					
state	Δ*E*	⟨*S*^2^⟩	metal	corrole	NO	radical character	Δ*E*^a^	radical character^b^
Fe[Cor]Cl								
^7^A′	15.0	12.03	4.21	1.54		1.00	12.2(14.3)	1.00
^7^A″	16.2	12.02	4.14	1.63		1.00	15.2(28.4)	1.00
^5^A′	10.1	6.82	4.07	–0.28		0.55	4.4(7.6)	0.60
^5^A″	6.6	6.05	2.69	1.05		1.00	11.4(12.5)	1.00
^3^A′	8.3	3.02	2.61	–0.87		0.84	13.8(26.1)	0.79
^3^A″	0.0	2.76	2.58	–0.77		0.47	0.0(0.0)	0.45(0.47)
^1^A′	18.7	1.49	1.06	–1.03		0.80	20.9(39.9)	0.73
Mn[Cor]Cl								
^6^A′	10.8	8.80	3.69	1.10		1.00	14.8	1.00
^6^A″	7.7	8.81	3.76	1.03		1.00	9.2	1.00
^4^A′	10.7	4.77	3.70	–0.91		0.83	15.0	0.86
^4^A″	0.0	4.48	3.62	–0.72		0.43	0.0	0.61(0.63)
^2^A′	24.2	1.98	1.94	–0.99		0.76	32.7	0.85
^2^A″	23.4	2.14	1.83	–0.88		0.99	39.4	0.89
Fe[Cor](NO)^c^								
^3^A	5.8	2.73	1.94	0.95	–0.89	1.00	17.5	1.00
^1^A	0.0	1.38	1.72	–0.84	–0.88	0.50	0.0	0.39
Ru[Cor](NO)								
^3^A	24.8	2.05	0.59	0.99	0.42	1.00	37.4	1.00
^1^A′	0.0	0.00	0.00	0.00	0.00	0.00	0.0	0.12
Fe[Cor]Ph								
^7^A′	28.9	12.0	4.25	1.50		1.00	37.0	1.00
^7^A″	31.3	12.0	4.19	1.58		1.00	39.9	1.00
^5^A′	20.4	6.56	4.05	–0.10		0.32	25.1	0.57
^5^A″	22.1	6.05	2.61	1.09		1.00	34.1	1.00
^3^A′	16.0	2.06	1.08	0.97		1.00	24.3	1.00
^3^A″	0.0	2.14	2.14	–0.10		0.00	0.0	small
^1^A′	14.8	1.02	1.13	–1.02		0.76	18.7	0.56
Mn[Cor]Ph								
^6^A′	25.2	8.80	3.65	1.16		1.00	49.2	1.00
^6^A″	24.4	8.80	3.72	1.09		1.00	42.5	1.00
^4^A′	26.3	3.81	1.97	1.04		1.00	43.1	1.00
^4^A″	0.0	3.91	3.08	–0.01		0.00	0.0	small
^2^A′	25.2	1.69	1.95	–0.95		0.68	38.7	0.71
^2^A″	24.3	1.81	1.93	–0.88		0.98	40.0	0.98
Mo[Cor]Cl_2_								
^4^A′	27.5	3.78	1.80	1.10		1.00	35.6	1.00
^4^A″	26.3	3.78	1.68	1.23		1.00	36.1	1.00
^2^A′	0.0	0.77	1.05	–0.10		0.01	0.0	0.20
^2^A″	27.4	0.77	0.01	0.99		1.00	32.9	1.00

a Numbers in brackets are CASPT2 results from ref (41).

b Calculated at the DMRG-CASSCF level. Values within parentheses were calculated with dichloromethane as the solvent.

c See ref (41).

Importantly, whereas B3LYP can generally correctly identify GSs, it affords only a qualitative description of excited-state energetics, routinely yielding different excited-state architectures relative to CASPT2. Indeed, the discrepancy between B3LYP and DMRG-CASPT2/CC energetics can range from near-zero to as large as a few tens of kilocalories per mole. For instance, in Fe[Cor](NO), B3LYP underestimates the ^1^A–^3^A gap (Δ*E*_1_) by ∼12 kcal/mol relative to DMRG-CASPT2/CC calculations, whereas the largest error of B3LYP is found for the ^6^A′–^4^A″ gap in Mn[Cor]Ph (24 kcal/mol). It has been shown that B3LYP has a tendency of overstabilizing HS as compared with LS states, so a modified functional with a smaller percentage of Hartree–Fock exchange (B3LYP*) has been proposed.^71,72^ Indeed, B3LYP* has been found to be one of the best functionals at describing the spin-state energetics of the spin-crossover complex Fe[salen](NO).^113^ We argue, however, that B3LYP* is not always better than B3LYP, and even when it is better, the improvement may be nominal (around 1 to 2 kcal/mol; see Tables S9–S15). Although the discrepancy between B3LYP and DMRG-CASPT2/CC may be discouraging to some, it is worth emphasizing that B3LYP predicts the correct GS in all complexes. Furthermore, we can find a linear correlation (with *R*^2^ ≈ 0.8) between B3LYP and DMRG-CASPT2/CC results (Figure S2).

The Fe[Cor]Cl molecule has been previously studied by Roos et al.,^40^ employing CASPT2 theory, ANO-RCC-TZVP basis sets, and, by current standards, small CAS(14,13) and CAS(14,14) active spaces. It is thus of some interest to compare our DMRG-CASPT2/CC results to the CASPT2 results of Roos et al.^40^ The general observation is that their CASPT2 relative energies are systematically higher than our DMRG-CASPT2/CC values. The smallest difference is only ∼1 kcal/mol for the ^5^A″–^3^A″ gap, whereas the largest difference is found for the ^1^A″–^3^A″ gap (19 kcal/mol)! This remarkable discrepancy probably indicates that the small active spaces employed by Roos et al.^40^ are insufficient. Another implication of an unsatisfactory active space is that the CAS(14,13) and CAS(14,14) active spaces can give very different results; for example, the ^3^A′–^3^A″ gap is predicted to be 18.6 kcal/mol with CAS(14,13) but 26.1 kcal/mol with CAS(14,14). Apparently, for comparing the relative energies of noninnocent states, the active space should include as many macrocycle π orbitals as possible so that subtle changes of corrole’s electron density can be captured.

### Quantification of Corrole Radical Character with B3LYP and DMRG-CASSCF Calculations

Table 2 presents B3LYP natural spin populations and the radical character of the corrole derived from both B3LYP and DMRG-CASSCF calculations. Again, we found that B3LYP yields qualitatively correct descriptions for all species, although, arguably, not quite for Mo[Cor]Cl_2_. Indeed, a linear correlation between B3LYP and DMRG-CASSCF radical character was found, as shown in Figure S1. For Fe[Cor]Cl, the GS is characterized as an *S* = 3*/*2 Fe(III) antiferromagnetically (AF) coupled to A′ Cor^•2–^. B3LYP predicts a combined corrole spin population of −0.77 and a modest corrole radical character of 0.47. The latter value is in excellent agreement with the radical character given by DMRG-CASSCF calculations (0.45). Both B3LYP and DMRG-CASSCF results indicate that the GS of Mn[Cor]Cl can be described as a quintet Mn(III) AF-coupled to A′ Cor^•2–^. B3LYP predicts that the corrole radical character of Mn[Cor]Cl is 0.43, slightly smaller than that of Fe[Cor]Cl. In contrast, the DMRG-CASSCF value (0.61) indicates that the corrole ring in Mn[Cor]Cl is considerably more “noninnocent” than that in the FeCl complex, which is somewhat surprising in view of the widespread occurrence of stable Mn(IV) complexes. That said, we certainly believe that the DMRG-CASSCF result is more reliable. For both Fe[Cor]Cl and Mn[Cor]Cl, the large corrole radical character reflects strong mixing between the metal (d_*z*^2^_) and the “porphyrin *a*_2*u*_-type”^114−118^ (a′) corrole orbitals.^6,13−19^

**Figure 1:**
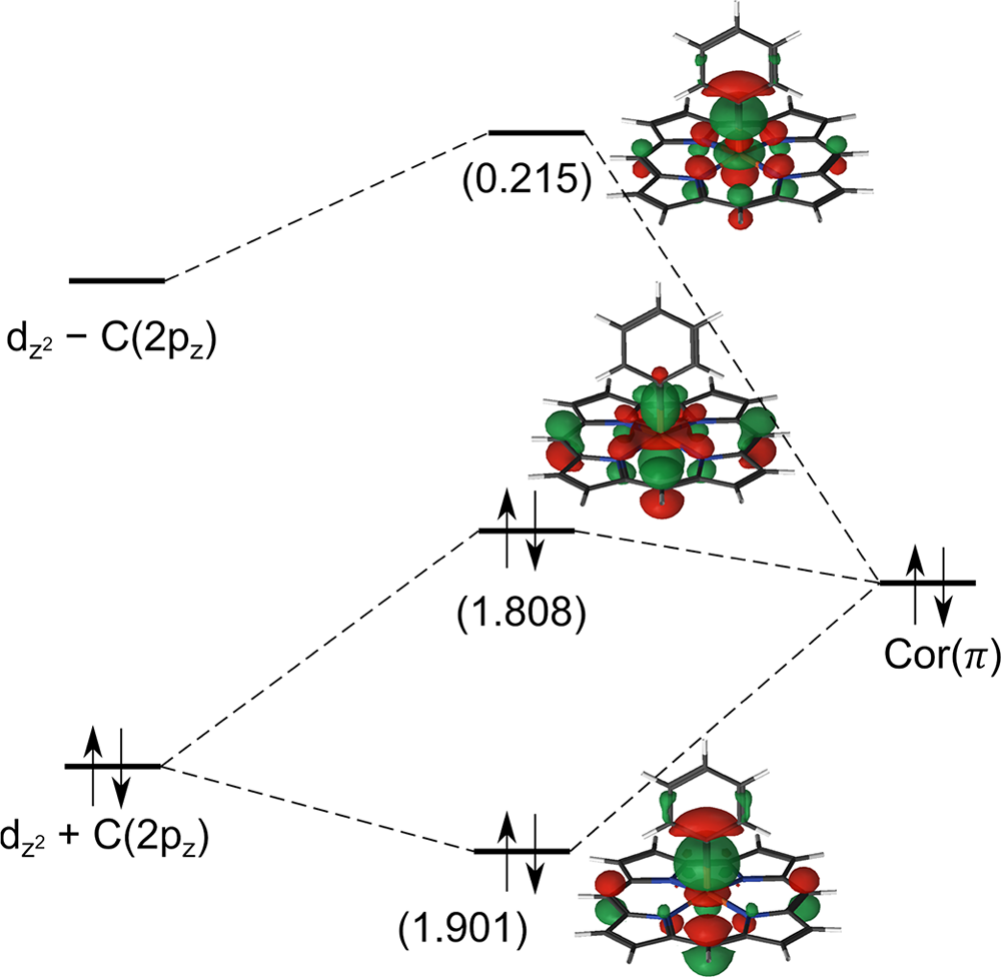
Mixing between the metal (d_*z*^2^_) orbital, phenyl–carbon (2p_*z*_) orbital, and “porphyrin *a*_2*u*_-type” corrole orbital in Fe[Cor]Ph. The numbers within parentheses are DMRG-CASSCF NOONs. The orbitals in Mn[Cor]Cl are similar.

In contrast to the chlorido complexes, the corrole ring in both phenyl complexes is essentially innocent. For B3LYP, the spin contamination is small—only 0.14 and 0.16 for Fe[Cor]Ph and Mn[Cor]Ph, respectively. The spin population on corrole is negligible, <0.1 in both complexes. DMRG-CASSCF wave functions show a mixing among three orbitals: the empty metal(d_*z*^2^_) orbital, the phenyl–carbon(2p_*z*_) orbital, and the “porphyrin *a*_2*u*_-type” or a′ corrole orbital (Figure 1). The former two mix strongly to yield the metal–phenyl σ bond. It is not trivial to determine the corrole radical character for these complexes because there are now three MOs involved. Nevertheless, on the basis of the NOONs and the shape of the orbitals, we expect the radical character to be small. The GS of Fe[Cor]Ph and Mn[Cor]Ph should be described as *S* = 1 Fe^IV^–Cor^3–^ and *S* = 3*/*2 Mn^IV^–Cor^3–^, respectively. Interestingly, these are the only states having a metal(IV) center and an innocent corrole. All excited states, in contrast, involve a metal(III) center (anti)ferromagnetically coupled to a Cor^•2–^ radical.

This study presents the first in-depth analysis of the electronic–structural differences between Fe[Cor](NO)^20,21,41,58^ and Ru[Cor](NO).^59^ Both exhibit singlet GSs but of very different character. As previously discussed,^20,41^ the Fe[Cor](NO) GS is an open-shell singlet involving an *S* = 1*/*2 {FeNO}^7^ moiety AF-coupled to a Cor^•2–^. The radical character of the corrole is significant — 0.50 with B3LYP and 0.39 with DMRG-CASSCF theory. B3LYP also yields a natural spin population of −0.84 on the corrole. In contrast, B3LYP indicates an *S* = 0 {RuNO}^6^ center and a closed-shell corrole for Ru[Cor](NO). All attempts to obtain a broken-symmetry {RuNO}^7^–Cor^•2–^ solution failed, suggesting a high energy for such an AF-coupled state. Another indication of a high-lying open-shell singlet is that the corresponding triplet state involving a {RuNO}^7^ center F-coupled to Cor^•2–^ lies ∼25 kcal/mol above the GS. DMRG-CASSCF theory confirms that the degree of noninnocence of corrole is minimal, with a radical character of only 0.12. Finally, it is worth noting that although corrole is innocent in Ru[Cor](NO), the nitrosyl is noninnocent in both the Fe and Ru complexes (*vide infra*).

For *S* = 1*/*2 Mo[Cor]Cl_2_, B3LYP calculations revealed that the Mo carries a spin population of 1.05; each Cl also carries a small positive spin population of 0.02, whereas the corrole carries a small negative spin population of around −0.10, suggesting a small amount of noninnocence. Experimentally, both telltale, crystallographically confirmed bond-length alternations^38^ and substituent-sensitive Soret maxima^39^ strongly suggest a noninnocent macrocycle in MoCl_2_ triarylcorrole derivatives. Surprisingly, the B3LYP NOON-based radical character for Mo[Cor]Cl_2_ turned out to be 0.01, that is, essentially negligible. In contrast, the CASSCF-DMRG radical character proved to be 0.2, confirming our earlier suggestion of a noninnocent corrole.

On the basis of the corrole radical character calculated with DMRG-CASSCF theory, we may conclude that three of the complexes studied, Mn[Cor]Cl, Fe[Cor]Cl, and Fe[Cor](NO), feature a noninnocent corrole. In contrast, Mn[Cor]Ph, Fe[Cor]Ph, and Ru[Cor](NO) are innocent, whereas Mo[Cor]Cl_2_ provides a fascinating example of a borderline case. Furthermore, we may rank the degree of noninnocent character of the corrole based on DMRG-CASSCF calculations, in decreasing order, as follows: Mn[Cor]Cl > Fe[Cor]Cl > Fe[Cor](NO) > Mo[Cor]Cl_2_ > Ru[Cor](NO) ≈ Fe[Cor]Ph ≈ Mn[Cor]Ph ≈ 0. Because these complexes are usually experimentally characterized in the presence of a solvent (e.g., dichloromethane, ε = 8.93), we also employed the polarizable continuum model (PCM)^119,120^ in DMRG-CASSCF calculations to account for solvation. By and large, the effects of solvation were found to be minimal (Table 2).

### Quantification of Corrole Radical Character with (R)CASCI

**Figure 2:**
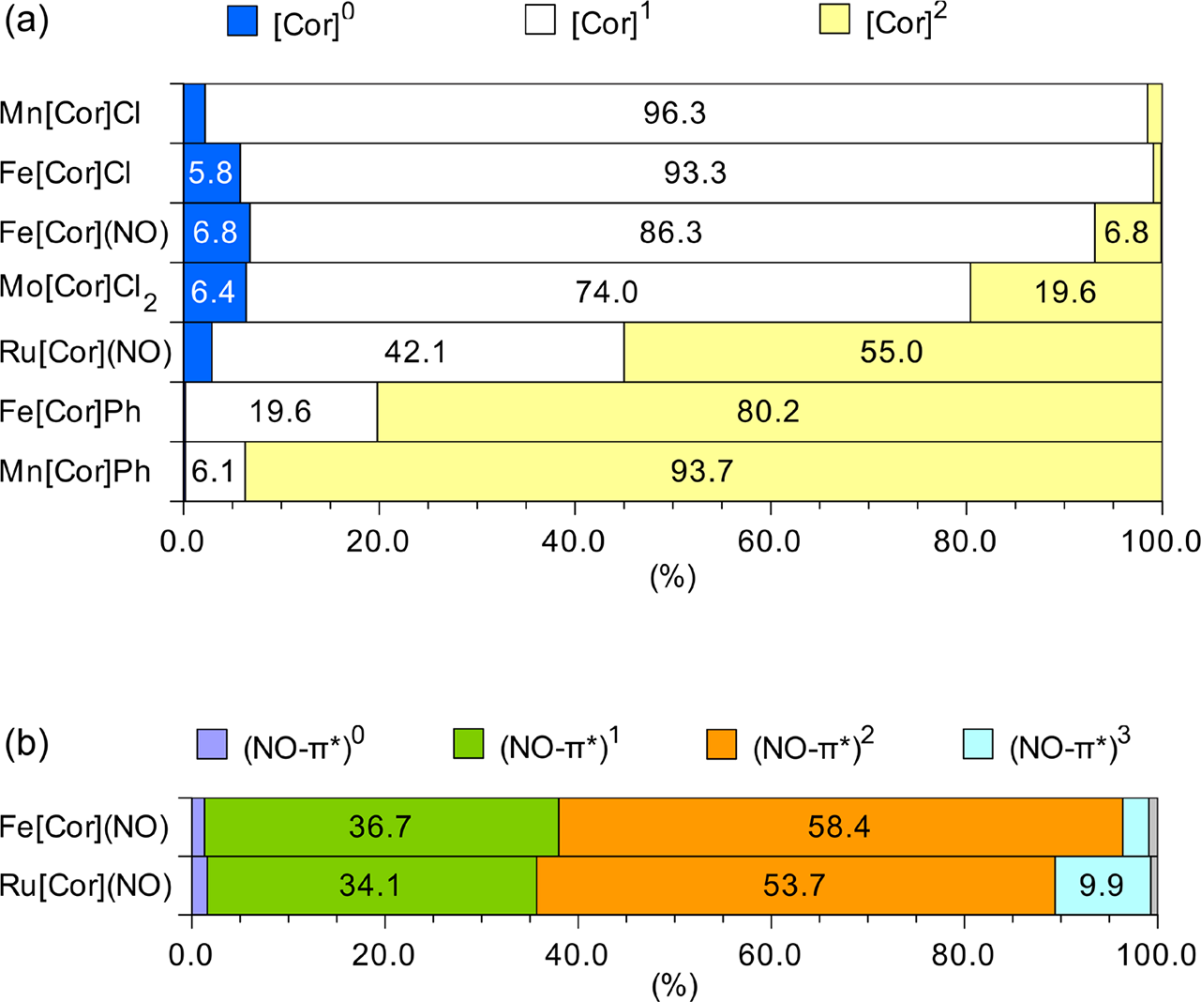
(a) Weights (in percentage) of dominant configurations based on [Cor]^*m*^ (*m* = 0, 1, 2) in CASCI wave functions. (b) Wave function of Fe[Cor](NO) and Ru[Cor](NO) can be also analyzed based on the weight of (NO-π*)^*m*^ (*m* = 0, 1, 2, 3) configurations. Only weights above 5% are labeled.

As in previous work,^40−42,45,50,53,55,105^ the GS wave functions can also be characterized in terms of localized molecular orbitals, referred to later as the “valence bond” wave function. To simplify the calculations and interpretations, we used minimal active spaces consisting of singly occupied metal d orbitals (see also Table 1), one corrole(π) orbital, and axial ligand (Cl, Ph, NO) orbitals that mix substantially with the metal d orbitals. The active spaces were thus CAS(4,4), CAS(5,5), CAS(6,5), CAS(7,6), CAS(6,6), CAS(6,6), and CAS(3,3) for Fe[Cor]Cl, Mn[Cor]Cl, Fe[Cor]Ph, Mn[Cor]Ph, Fe[Cor](NO), Ru[Cor](NO), and Mo[Cor]Cl_2_, respectively. The orbitals were localized from DMRG-CASSCF orbitals, and CASCI calculations were performed on top of these localized orbitals. Given our interest in the radical character of the corrole, the wave functions were decomposed into resonance structures (d)^*n*^[Cor]^*m*^, with *m* = 0, 1, and 2. The weights of dominant configurations in the CASCI wave functions are shown in Figure 2 and Table S1. The “valence bond” wave function consists of two parts: covalent ([Cor]^1^ configuration) and ionic ([Cor]^0^ and [Cor]^2^ configurations). Therefore, the weight of the [Cor]^1^ configuration does not equal, but does correlate with, the radical character of the corrole. The GS of Mn[Cor]Cl is dominated by the d^4^[Cor]^1^ configuration (up to 96%) and has negligible contributions of the d^5^[Cor]^0^ and d^3^[Cor]^2^ configurations (<2%). The results corroborate the finding that the radical character of the corrole in Mn[Cor]Cl (0.61) is the highest among the complexes studied. Likewise, the leading configuration in the GS wave function of Fe[Cor]Cl is [Cor]^1^ (93.3%).

In contrast with the chlorido complexes, Mn[Cor]Ph and Fe[Cor]Ph exhibit a [Cor]^2^ leading configuration, with weights of 93.7 and 80.2%, respectively. In the latter complex, the contribution of (d, Ph-σ)^7^[Cor]^1^ is not negligible (19.6%), suggesting that the corrole ring is not fully closed-shell. Nevertheless, on the basis of a small corrole radical character value (Table 2), these two complexes may both be described as M^IV^–Cor^3–^. The large contribution of the [Cor]^1^ configuration thus signifies the advantage of the configuration analysis as compared with the NOON analysis; that is, the weights of the leading configurations are highly sensitive to the radical character of corrole.

As previously mentioned,^41^ the configuration analysis confirms the {FeNO}^7^–Cor^•2–^ description of Fe[Cor](NO), with the leading configuration (d, NO-π*)^7^[Cor]^1^ accounting for >80%. In Ru[Cor](NO), the GS wave function is a mixture of two configurations, (d, NO-π*)^7^[Cor]^1^ (42%) and (d, NO-π*)^6^[Cor]^2^ (55%). The contribution of the latter configuration is somewhat larger, showing that this complex is “more” closed-shell than open-shell. Another way to look at the GS wave function of Fe[Cor](NO) and Ru[Cor](NO) is to sum the weights of all (d, Cor)^*n*^(NO-π*)^*m*^ configurations, with *m* = 0, 1, 2, and 3 (Figure 2b). This approach allows us to analyze the nature of the nitrosyl group. It was shown^41^ that NO binds primarily as NO^–^ or NO because the leading configurations are (d, Cor)^6^(NO-π*)^2^ (∼60%) and (d, Cor)^7^(NO-π*)^1^ (∼37%). As in Fe[Cor](NO), the nitrosyl in Ru[Cor](NO) binds as either NO^–^ or NO with comparable contributions (53.7 and 34.1%, respectively). The nature of the metal–NO bond in these complexes is in agreement with previous findings by Radoń et al.^55^

The configuration analysis confirms that a noninnocent description for corrole Mo[Cor]Cl_2_ as the leading configuration is (d)^2^[Cor]^1^. As expected, however, its weight (74%) is lower than that of the analogous [Cor]^1^ configurations of Mn[Cor]Ph (96%), Fe[Cor]Ph (93%), and Fe[Cor](NO) (86%) but substantially higher relative to the analogous configurations of Mn[Cor]Ph (6%), Fe[Cor]Ph (20%), and Ru[Cor](NO) (42%). These results nicely confirm our previous conclusion (see above) that Mo[Cor]Cl_2_ is best viewed as borderline noninnocent.

As previously discussed, the configuration analysis gives results that are more sensitive to the radical character of corrole. Thus we may rank the systems studied according to the weight of the [Cor]^1^ configuration: Mn[Cor]Cl > Fe[Cor]Cl > Fe[Cor](NO) > Mo[Cor]Cl_2_ > Ru[Cor](NO) > Fe[Cor]Ph > Mn[Cor]Ph. This order is in essentially perfect agreement with the order based on the DMRG-CASSCF corrole radical character: Mn[Cor]Cl > Fe[Cor]Cl > Fe[Cor](NO) > Mo[Cor]Cl_2_ > Ru[Cor](NO) ≈ Mn[Cor]Ph ≈ Fe[Cor]Ph ≈ 0.

**Figure 3:**
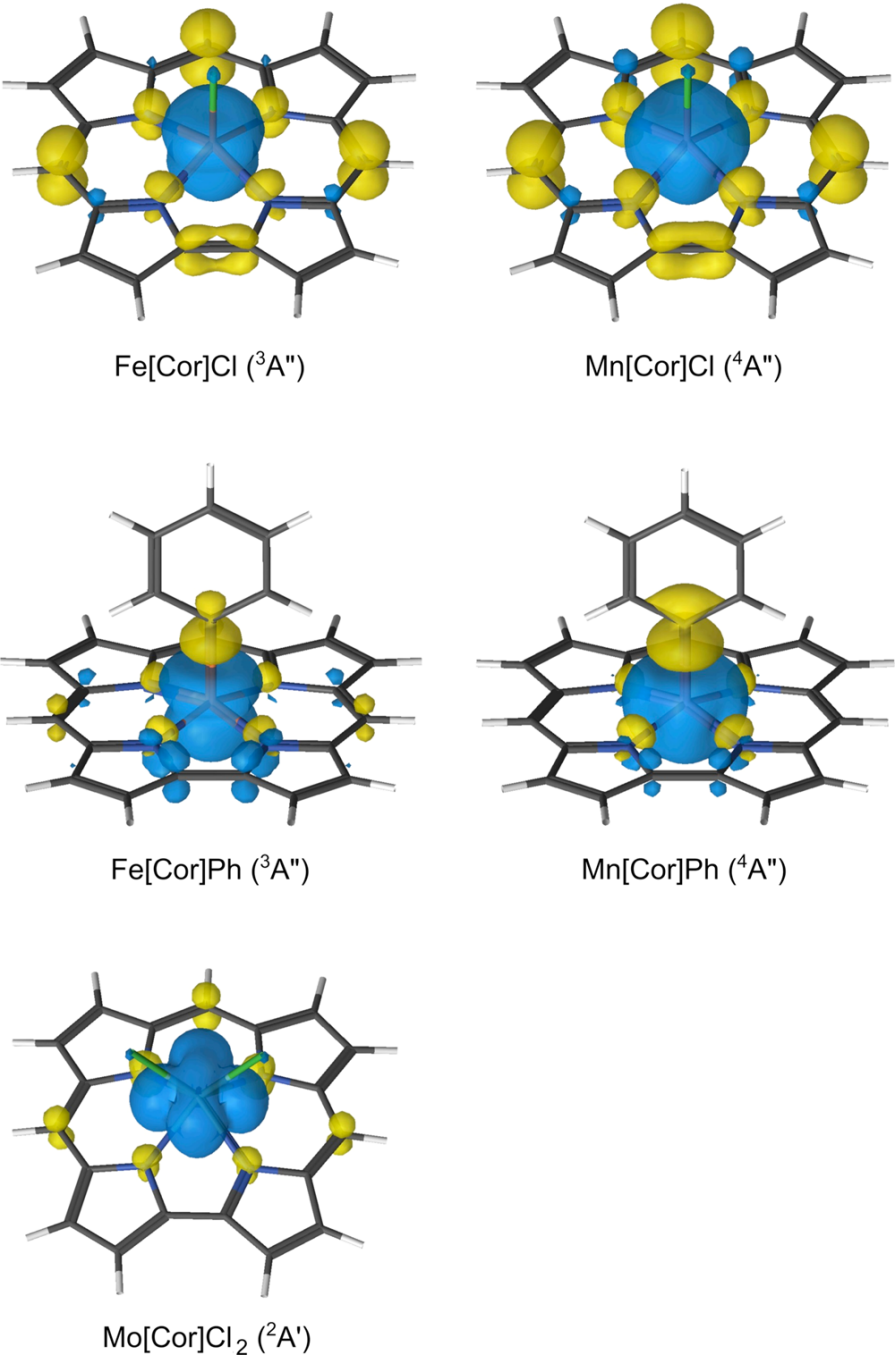
RASCI spin density of Fe[Cor]Cl, Mn[Cor]Cl, Fe[Cor]Ph, Mn[Cor]Ph, and Mo[Cor]Cl_2_. The contour values are ±0.002 e/au^3^. Blue, positive spin density; yellow, negative spin density.

Another straightforward way to analyze corrole radical character is to visualize the DMRG-CASSCF spin density. Unfortunately, this functionality has not been implemented in the OpenMolcas-CheMPS2 interface. We therefore calculated the spin density using RASCI on top of DMRG-CASSCF orbitals. These results are depicted in Figure 3. Obviously, spin density plots for singlet Fe[Cor](NO) and Ru[Cor](NO) are not relevant (because the spin density at the CASSCF level must be zero everywhere). On the contrary, in both Fe[Cor]Cl and Mn[Cor]Cl, the corrole is noninnocent, which may be seen most clearly by the accumulation of negative spin density at the *meso* carbons. These spin density plots are in good, qualitative agreement with those obtained from DFT.^6,13−19^ A close inspection of the RASCI spin density reveals that corrole in Mn[Cor]Cl has a higher negative spin accumulation than that in Fe[Cor]Cl and, again, that the corrole radical character is slightly higher in Mn[Cor]Cl than in Fe[Cor]Cl. Note that we also found small blobs of both positive and negative spin density on the corrole in Fe[Cor]Ph. In Mn[Cor]Ph, in contrast, the spin density on the corrole is negligible. For Mo[Cor]Cl_2_, the spin density pattern is qualitatively similar to that of Mn[Cor]Cl and Fe[Cor]Cl, but the amount of spin density on the corrole is much smaller than that in either of the two molecules. These results are all in excellent accord with those obtained with configuration analysis (Figure 2).

## Concluding Remarks

The electronic structures of seven potentially noninnocent middle transition metal corroles have been analyzed in unprecedented detail with DMRG-CASPT2 calculations. The results have afforded a host of insights into the performance of hybrid DFT (B3LYP), excited-state energetics, and ground-state ligand noninnocence, some of the key highlights being as follows.

The ground-state electronic structures of Fe–Cl/Ph and Mn–Cl/Ph corroles have been thought of as entirely analogous until now.^6^ Whereas that qualitative conclusion remains unchanged, substantial quantitative differences between the two metals have become apparent. Thus, for both chlorido complexes Mn[Cor]Cl and Fe[Cor]Cl, the ferromagnetically coupled M^III^–Cor^•2–^ state is some 10 kcal/mol higher (DMRG-CASPT2 excitation energies) than the antiferromagnetically coupled ground state. In the FeCl case, however, the ferromagnetically coupled state does not correspond to the first excited state, which (at an energy of only 4.4 kcal/mol above the ground state) instead involves a high-spin (locally *S* = 5/2) Fe(III) center antiferromegnatically coupled to a corrole A′ radical.

Substantial differences in excited-state energetics are also observed between Mn[Cor]Ph and Fe[Cor]Ph. Thus the two lowest excited states of Fe[Cor]Ph, which correspond to an intermediate-spin *S* = 3/2 Fe(III) center antiferromagnetically coupled to a corrole A″ radical (overall *S* = 1) and a high-spin *S* = 5/2 Fe(III) center antiferromagnetically coupled to a corrole A′ radical (overall *S* = 2), are about 24 to 25 kcal/mol (DMRG-CASPT2 excitation energies) above the essentially low-spin Fe(IV) ground state. In contrast, the analogous excited states of Mn[Cor]Ph are much higher, by >40 kcal/mol, relative to the essentially Mn(IV) ground state. Fascinatingly, the much higher excitation energies in the MnPh case are in qualitative accord with dramatically higher electrochemical HOMO–LUMO gaps (defined as the algebraic difference between the oxidation and reduction potentials) of MnPh corroles relative to analogous FePh corroles.^14^

The present study provides some of the first definitive insights into the electronic–structural differences between Fe[Cor](NO) and Ru[Cor](NO), a point on which we had only speculated about until now.^59^ We can now confirm that whereas ground-state Fe[Cor](NO) is best described as a locally *S* = 1/2 {FeNO}^7^ unit antiferromagnetically coupled corrole A′ radical, Ru[Cor](NO) is simply {RuNO}^6^–Cor^3–^, that is, with an innocent corrole macrocycle. Understandably, whereas the ferromagnetically coupled *S* = 1{FeNO}^7^–Cor^•2–^ state of Fe[Cor](NO) is only ∼17.5 kcal/mol higher than the antiferromagnetically coupled, *S* = 0 ground state, the analogous triplet state of Ru[Cor](NO) is higher by a far larger margin (37.4 kcal/mol) relative to the ground state. In the same vein, Mo[Cor]Cl_2_ exhibits a doublet-quartet gap of 36.1 kcal/mol. The results on Ru[Cor](NO) and Mo[Cor]Cl_2_ afford rare, quantitative insight into the relative energetics of metal–ligand spin coupling for 3d versus 4d transition metals.

Finally, several metrics derived from both B3LYP and DMRG-CASSCF calculations, including NOON values, were used to assess and compare the radical character (or noninnocence) of the corrole ligand for the seven complexes studied. The metrics proved mutually consistent and yielded the following ordering for corrole radical character, Mn[Cor]Cl > Fe[Cor]Cl > Fe[Cor](NO) > Mo[Cor]Cl_2_ > Ru[Cor](NO) ≈ Mn[Cor]Ph ≈ Fe[Cor]Ph ≈ 0, the first such ordering for metallocorroles.

One of the more interesting findings in this connection is that Fe[Cor](NO), while substantially noninnocent, is less so than FeCl (or MnCl) corrole, which is consistent with X-ray absorption spectra showing a less intense 1s → 3d absorption for an FeNO corrole relative to an analogous FeCl corrole.^14,19^ In contrast, the corrole in Ru[Cor](NO) is almost perfectly innocent, confirming previous speculations to that effect.^59^

Finally, our characterization of Mo[Cor]Cl_2_ as a borderline noninnocent species is also of unusual interest. It represents the point of onset where ligand noninnocence starts to manifest itself via multiple experimental metrics, such as the molecular structure (X-ray crystallography) and optical spectra. The identification and detailed characterization of such borderline species for ligand systems other than corrole may lead to further advances in our understanding of ligand noninnocence.

## Supporting Information Available

Optimized Cartesian coordinates. Correlation between DMRG-CASSCF and B3LYP radical character of corrole. Correlation between DMRG-CASPT2/CC and B3LYP relative energies. Weights (in percentage) of dominant configurations in CASCI wave functions in the gas phase and dichloromethane solvent. Total and relative energies calculated with B3LYP, DMRG-CASSCF, DMRG-CASPT2, and CCSD(T). Root-mean-square deviation (rmsd) between B3LYP and B3LYP* geometries. Comparison between B3LYP and experimental geometries. Active spaces of the states and active orbitals of the ground states (PDF)
