# Supplementary material for: A DMRG/CASPT2 Investigation of Metallocorroles: Quantifying Ligand Noninnocence in Archetypal 3d and 4d Element Derivatives
Source: JACS Au. 2021 Oct 21;1(12):2303–14. doi: 10.1021/jacsau.1c00417 (PMC8717376; doi:10.1021/jacsau.1c00417)
Supplement: Supplementary file 1 — au1c00417_si_001.pdf [file au1c00417_si_001.pdf]

Supporting Information

A DMRG/CASPT2 Investigation of Metallocorroles: Quantifying Ligand Noninnocence in Archetypal 3d and 4d Element Derivatives

Quan Manh Phung,*[,†,‡] Yasin Muchammad,[†] Takeshi Yanai,[†,‡] and Abhik Ghosh*[,¶]

†*Department of Chemistry, Graduate School of Science, Nagoya University, Furo-cho, Chikusa-ku, Nagoya, Aichi, 464-8602, Japan*

‡*Institute of Transformative Bio-Molecules (WPI-ITbM), Nagoya University, Furo-cho, Chikusa-ku, Nagoya, Aichi, 464-8602, Japan*

¶*Department of Chemistry, UiT-The Arctic University of Norway, N-9037 Tromsø, Norway*

E-mail: quan.phung@chem.nagoya-u.ac.jp (QMP); abhik.ghosh@uit.no (AG)

Table of Contents

Content	Page
A. List of states that are not minima on the potential energy surfaces	S2
B. Figures S1-S9	S3
C. Tables S1-S17	S11
D. Optimized B3LYP Cartesian coordinates (Å)	S18

A. List of states that are not minima on the potential energy surfaces

- Fe[Cor]Cl: $^{7}A'$, $^{1}A'$
- Mn[Cor]Cl: $^{2}A'$, $^{6}A'$
- Fe[Cor]Ph: $^{1}A'$, $^{3}A'$, $^{5}A''$, $^{7}A'$, $^{7}A''$
- Mn[Cor]Ph: $^{2}A'$, $^{4}A'$, $^{6}A'$, $^{6}A''$
- Mo[Cor]Cl_2: $^{4}A'$, $^{4}A''$

B. Figures S1-S9

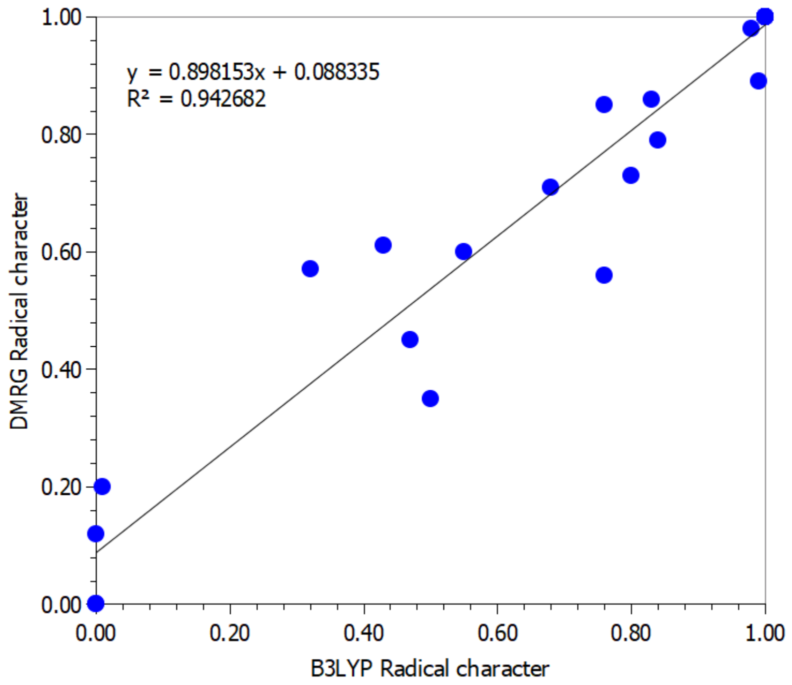

Figure S1. Correlation between DMRG and B3LYP radical character of corrole.

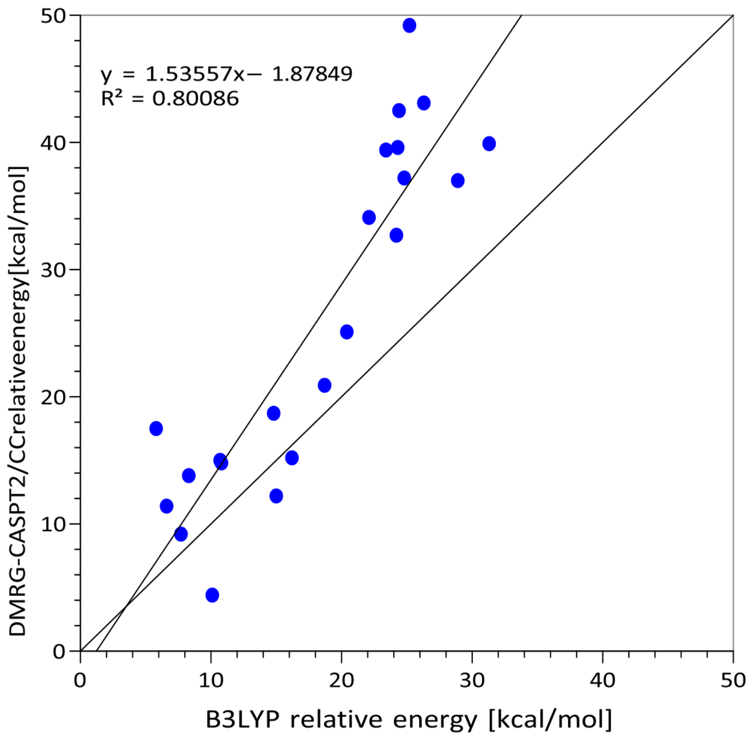

Figure S2. Correlation between DMRG-CASPT2/CC and B3LYP relative energies.

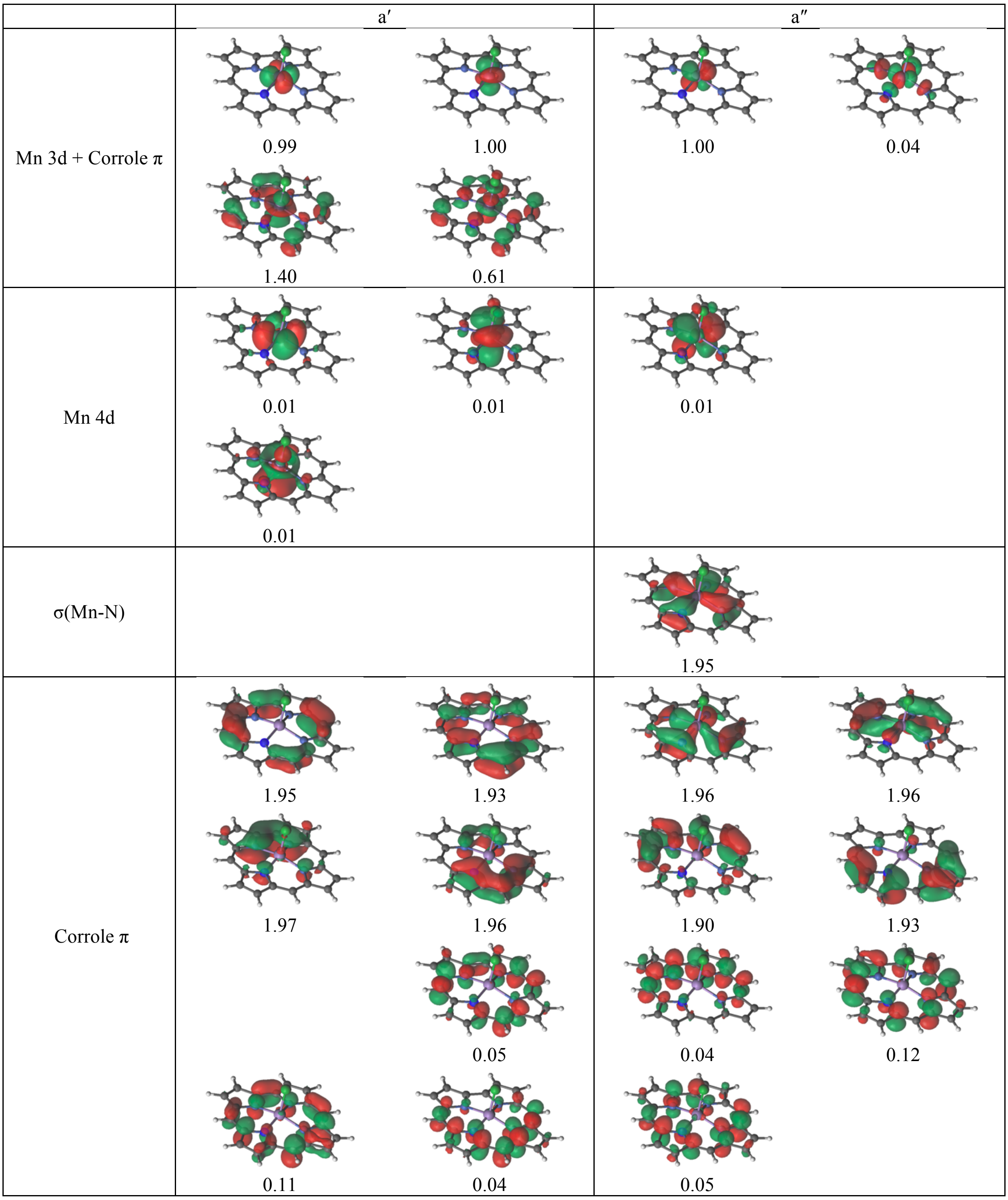

Figure S3. Active natural orbitals and their occupation numbers (NOONs) of Mn[Cor]Cl ground state $^4A''$.

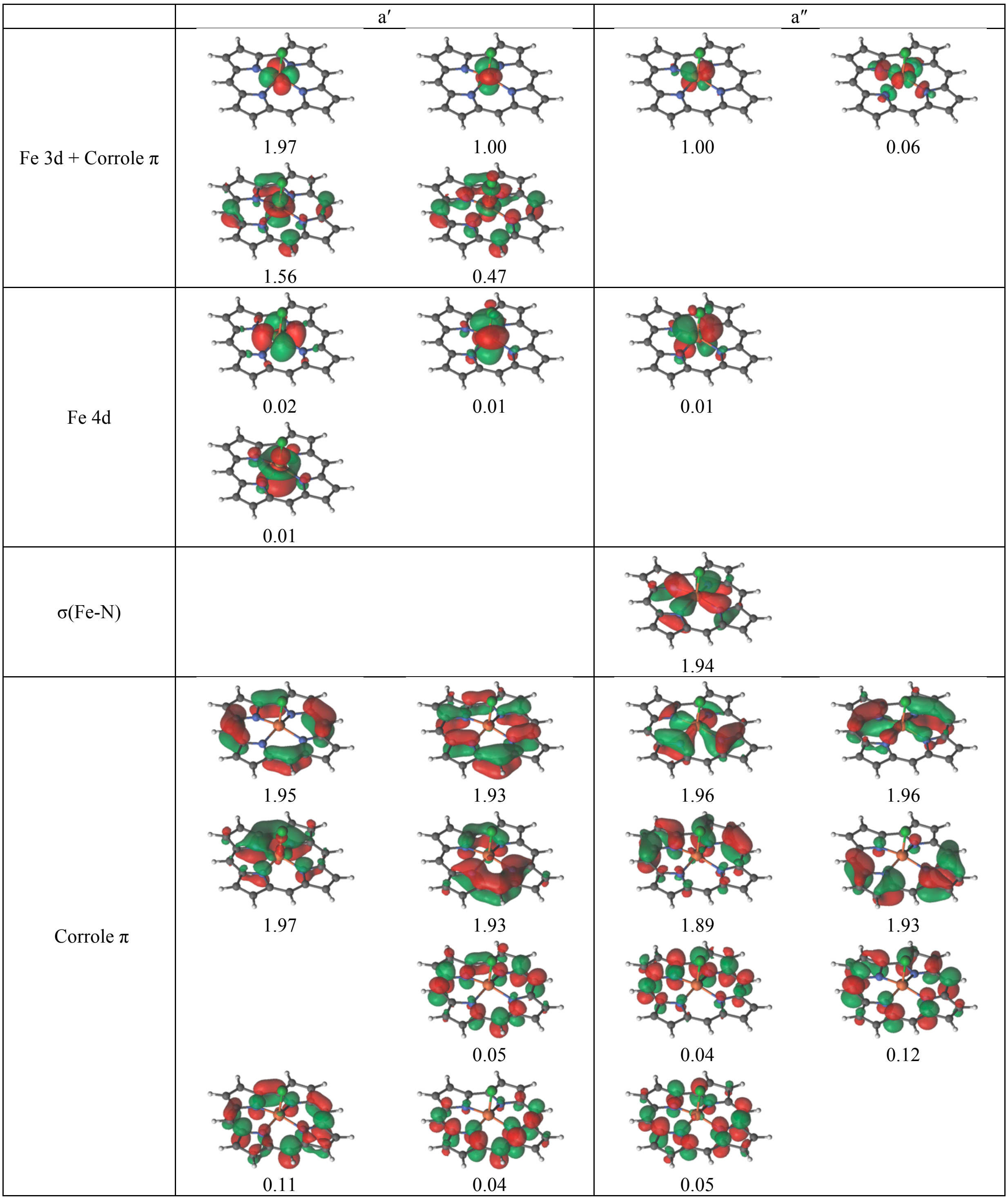

Figure S4. Active natural orbitals and their occupation numbers (NOONs) of Fe[Cor]Cl ground state $^{3}A''$.

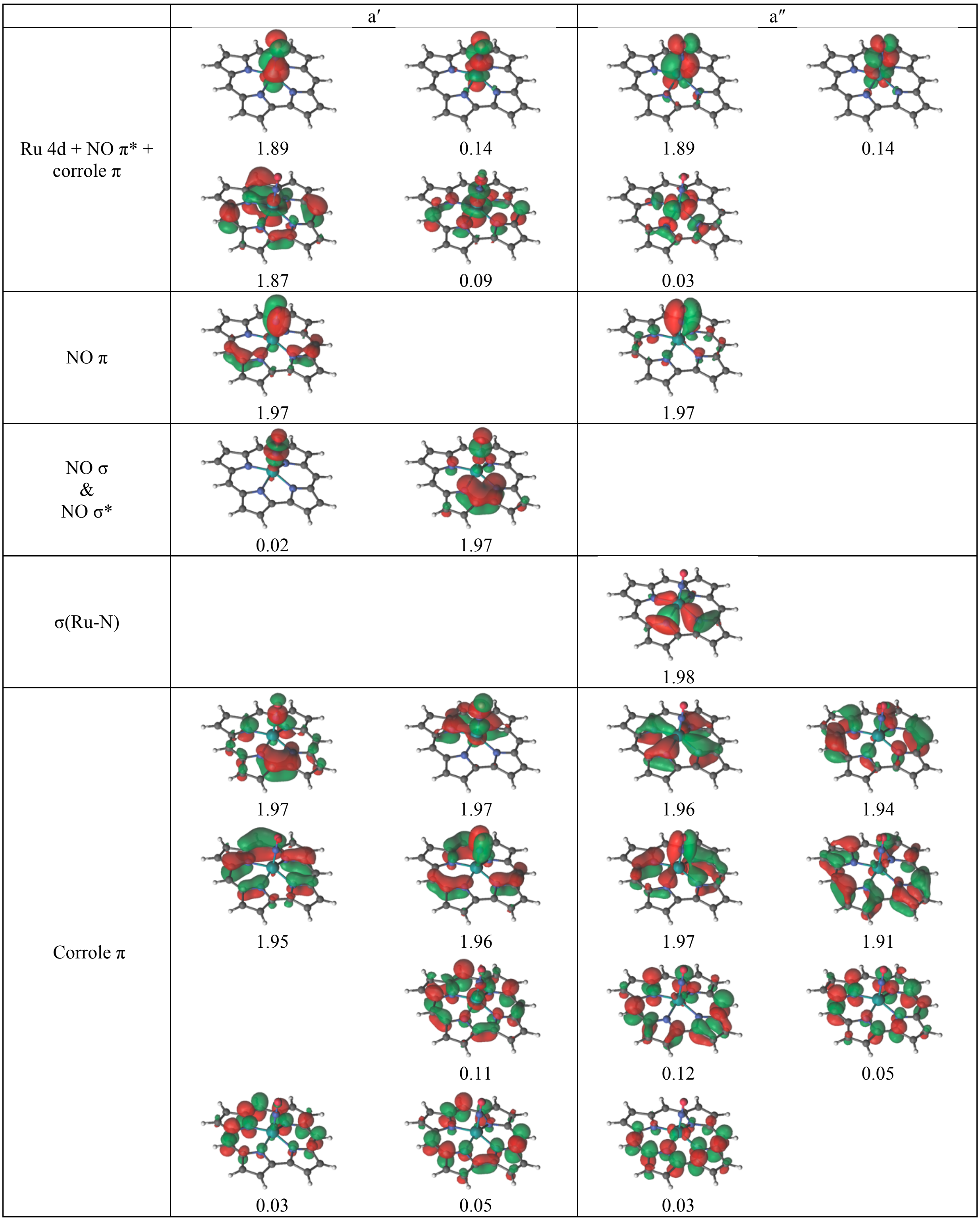

Figure S5. Active natural orbitals and their occupation numbers (NOONs) of Ru[Cor]NO ground state $^1A'$.

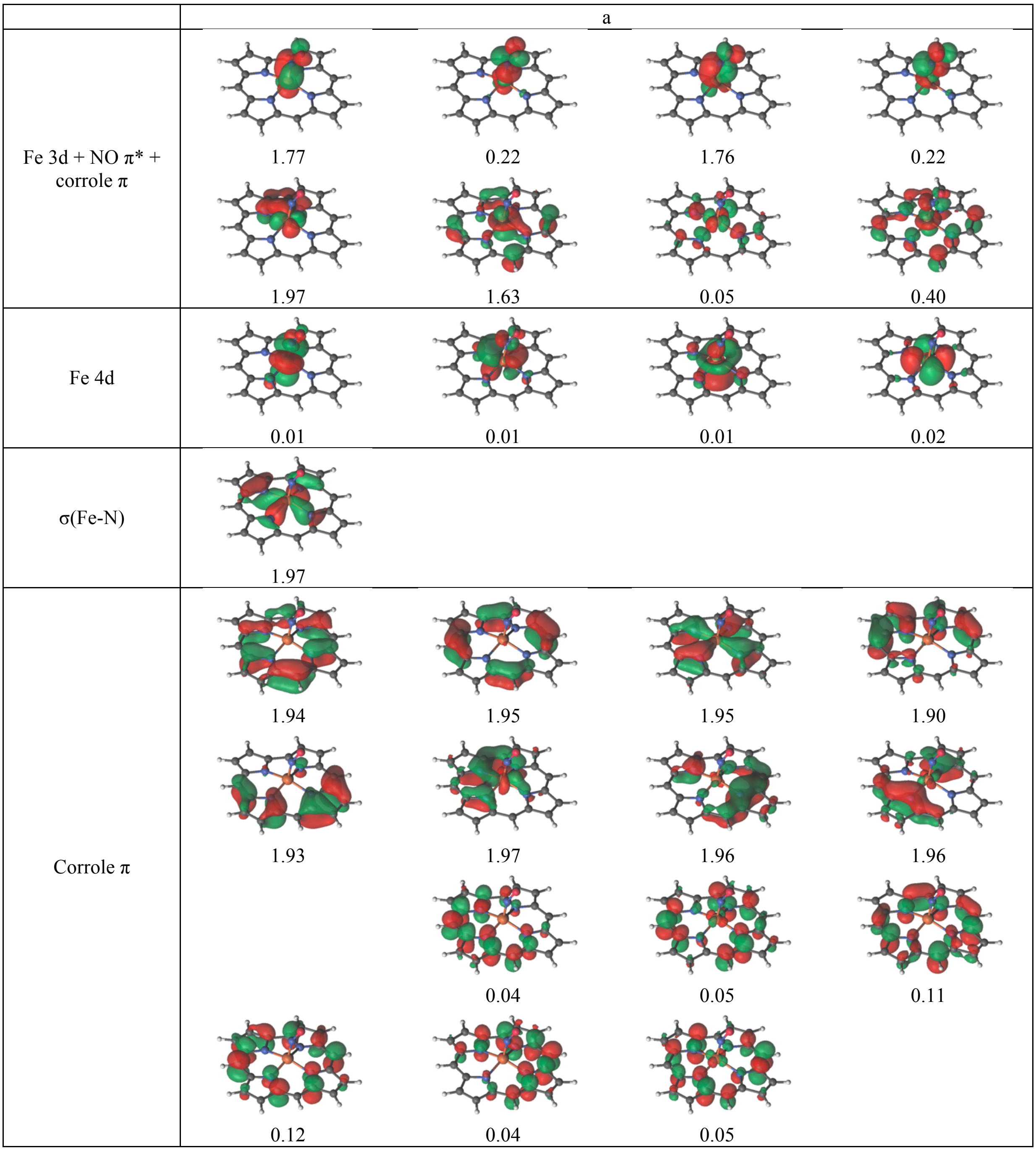

Figure S6. Active natural orbitals and their occupation numbers (NOONs) of Fe[Cor]NO ground state ^{1}A.

	a′		a″	
Fe 3d	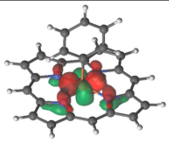 1.97	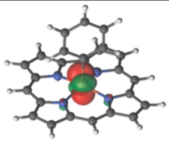 1.01	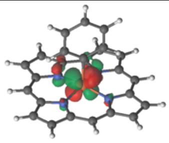 1.03	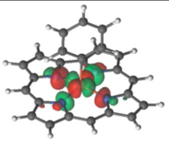 0.06
		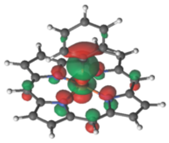 0.21		
Fe 4d	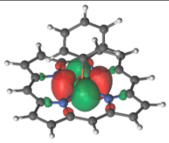 0.02	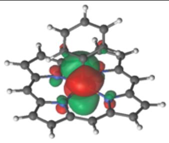 0.01	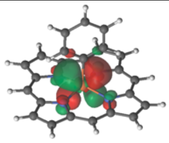 0.01	
	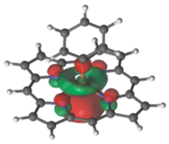 0.01			
σ(Fe-C)	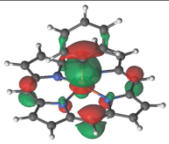 1.95			
σ(Fe-N)			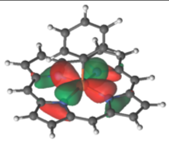 1.95	
Corrole π	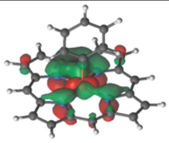 1.97	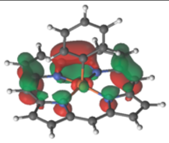 1.97	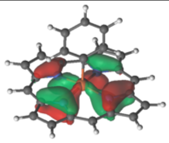 1.96	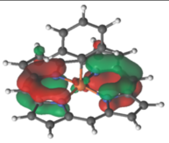 1.96
	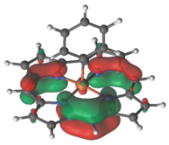 1.96	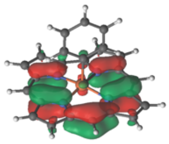 1.95	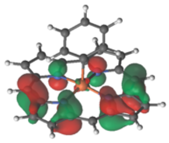 1.94	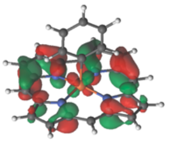 1.89
	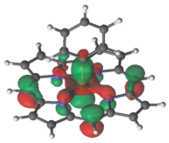 1.81	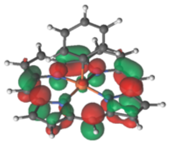 0.11	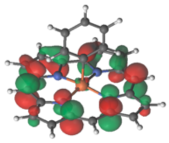 0.13	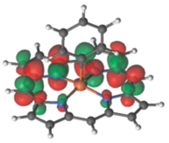 0.03
	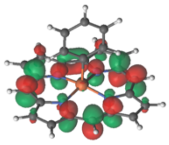 0.05	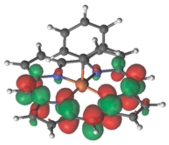 0.04	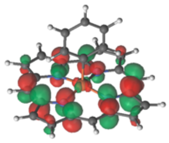 0.05	

Figure S7. Active natural orbitals and their occupation numbers (NOONs) of Fe[Cor]Ph ground state $^3A''$.

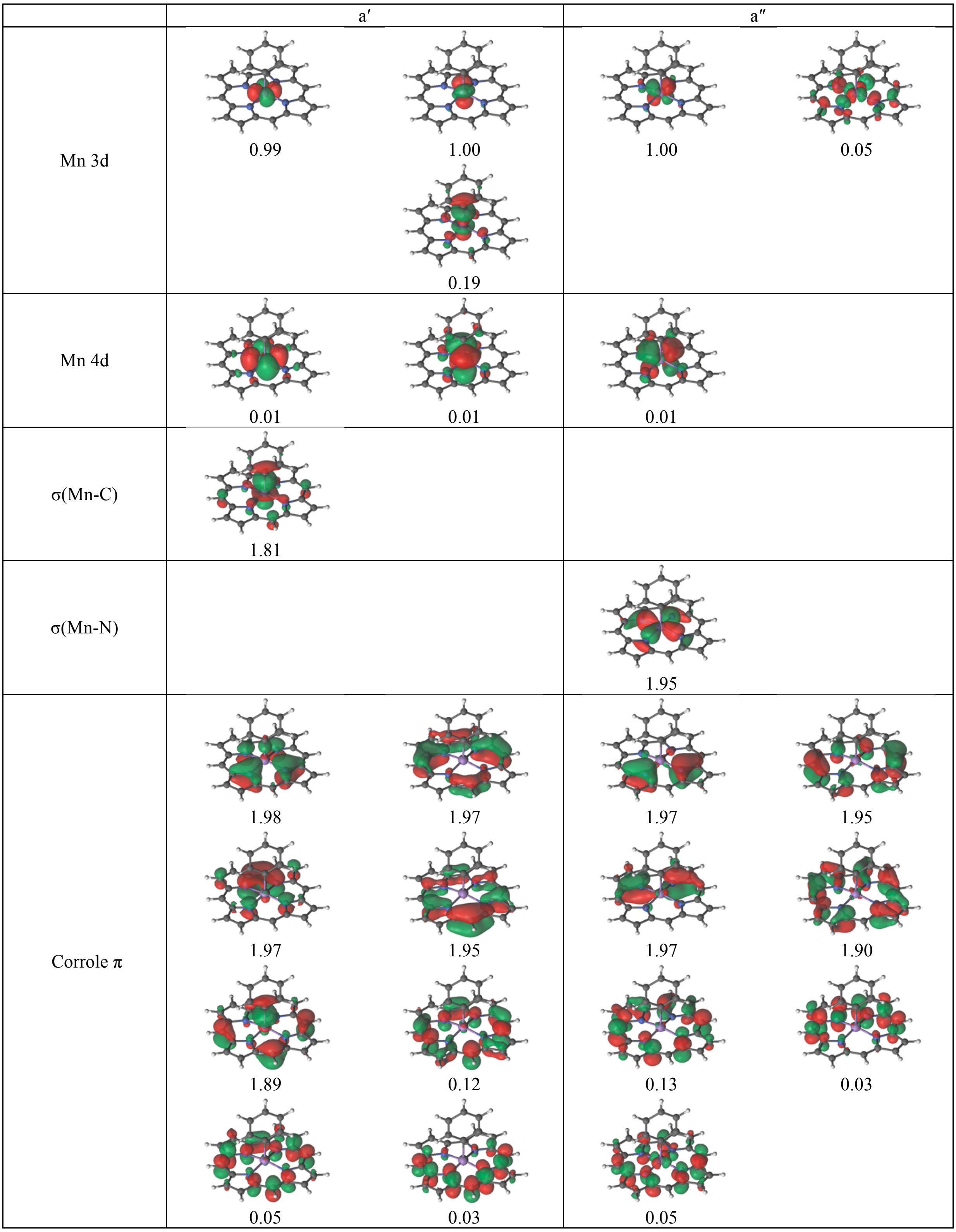

Figure S8. Active natural orbitals and their occupation numbers (NOONs) of Mn[Cor]Ph ground state $^4A''$.

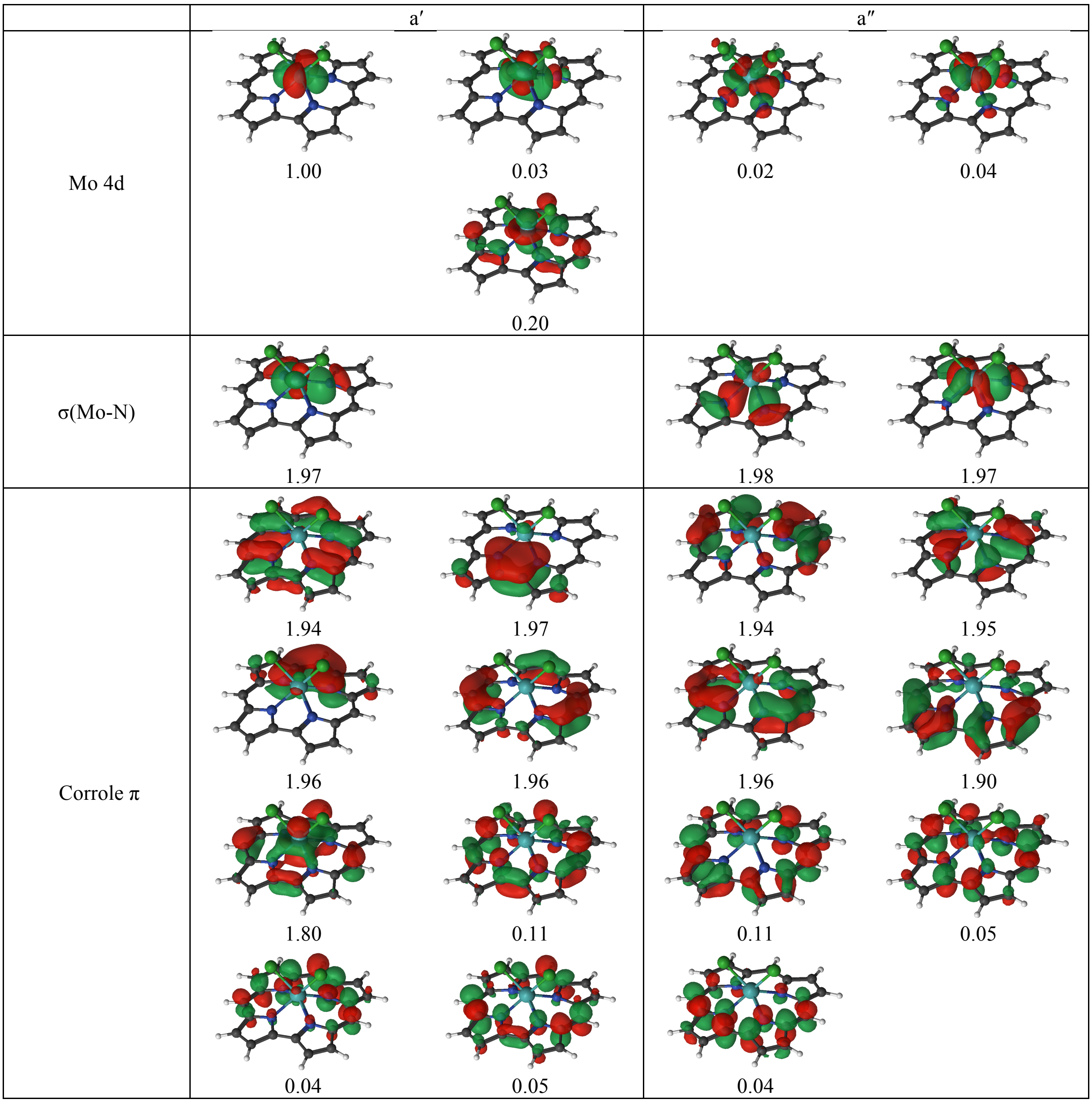

Figure S9. Active natural orbitals and their occupation numbers (NOONs) of $Mo[Cor]Cl_2$ ground state $^2A'$.

C. Tables S1-S17

Table S1. Weights (in percentage) of dominant configurations in CASCI wavefunctions in dichloromethane solvent

State	Configuration	Weight (%)
Fe[Cor]Cl ^{3}A″	(d)^6[Cor]^0	5.5
	(d)^5[Cor]^1	93.8
	(d)^4[Cor]^2	0.7
Mn[Cor]Cl ^{4}A″	(d)^5[Cor]^0	1.8
	(d)^4[Cor]^1	96.8
	(d)^3[Cor]^2	1.2
Fe[Cor]Ph ^{3}A″	(d, Ph□)^8[Cor]^0	0.2
	(d, Ph□)^7[Cor]^1	20.5
	(d, Ph□)^6[Cor]^2	79.2
Mn[Cor]Ph ^{4}A″	(d, Ph□)^7[Cor]^0	0.0
	(d, Ph□)^6[Cor]^1	6.1
	(d, Ph□)^5[Cor]^2	93.7
Fe[Cor]NO ^{1}A	(d, NO□*)^8[Cor]^0	7.3
	(d, NO□*)^7[Cor]^1	86.8
	(d, NO□*)^6[Cor]^2	5.1
Ru[Cor]NO ^{1}A′	(d, NO□*)^8[Cor]^0	2.0
	(d, NO□*)^7[Cor]^1	39.6
	(d, NO□*)^6[Cor]^2	57.9
Mo[Cor]Cl_2 ^{2}A′	(d)^3[Cor]^0	7.4
	(d)^2[Cor]^1	76.5
	(d)^1[Cor]^2	16.0
Fe[Cor]NO ^{1}A	(d, Cor)^8(NO□*)^0	1.3
	(d, Cor)^7(NO□*)^1	36.7
	(d, Cor)^6(NO□*)^2	58.4
	(d, Cor)^5(NO□*)^3	2.7
Ru[Cor]NO ^{1}A′	(d, Cor)^8(NO□*)^0	1.6
	(d, Cor)^7(NO□*)^1	33.8
	(d, Cor)^6(NO□*)^2	54.1
	(d, Cor)^5(NO□*)^3	9.8

Table S2. Total Energies (in au) of Mn[Cor]Cl

	B3LYP[a]	B3LYP*[a]	DMRG[b]	CASPT2		CCSD(T)	
				3s3p[c]	0s0p[d]	3s3p[c]	0s0p[d]
$^{2}A'$	-2561.452769	-2560.585981	-2562.917869	-2567.337835	-2566.917398	-2563.190230	-2562.771807
$^{2}A''$	-2561.453988	-2560.586454	-2562.887648	-2567.332309	-2566.913640	-2563.184269	-2562.772856
$^{4}A'$	-2561.474257	-2560.603953	-2562.964161	-2567.368759	-2566.948208	-2563.193298	-2562.777519
$^{4}A''$	-2561.491288	-2560.623518	-2562.965266	-2567.392660	-2566.971769	-2563.219323	-2562.803190
$^{6}A'$	-2561.474057	-2560.603533	-2562.964273	-2567.369110	-2566.948607	-2563.193340	-2562.777595
$^{6}A''$	-2561.479005	-2560.608720	-2562.958795	-2567.377225	-2566.957750	-2563.197811	-2562.782243

[a]def2-TZVP basis set. [b]m = 4000. [c]With metal 3s and 3p electrons correlated. [d]Without 3s and 3p electrons correlated.

Table S3. Total Energies (in au) of Fe[Cor]Cl

	B3LYP[a]	B3LYP*[a]	DMRG[b]	CASPT2		CCSD(T)	
				3s3p[c]	0s0p[d]	3s3p[c]	0s0p[d]
$^{1}A'$	-2674.163055	-2673.290889	-2676.941186	-2681.403450	-2680.981466	-2677.231447	-2676.804186
$^{3}A'$	-2674.179638	-2673.305364	-2676.958731	-2681.414223	-2680.992844	-2677.229620	-2676.802343
$^{3}A''$	-2674.192894	-2673.320387	-2676.962495	-2681.436779	-2681.014629	-2677.248693	-2676.821287
$^{5}A'$	-2674.176876	-2673.299018	-2676.999061	-2681.431833	-2681.013292	-2677.204921	-2676.783191
$^{5}A''$	-2674.182330	-2673.308170	-2676.949499	-2681.417449	-2680.996708	-2677.230578	-2676.803470
$^{7}A'$	-2674.168974	-2673.290442	-2676.990556	-2681.417761	-2681.001317	-2677.193163	-2676.771907
$^{7}A''$	-2674.167121	-2673.288522	-2676.997873	-2681.414075	-2680.996221	-2677.189098	-2676.767541

[a]def2-TZVP basis set. [b]m = 4000. [c]With metal 3s and 3p electrons correlated. [d]Without 3s and 3p electrons correlated.

Table S4. Total Energies (in au) of Ru[Cor]NO

	B3LYP[a]	B3LYP*[a]	DMRG[b]	CASPT2		CCSD(T)	
				4s4p[c]	4s4p[d]	4s4p[c]	4s4p[d]
$^{1}A'$	-1175.121120	-1174.440204	-5601.490759	-5606.171999	-5605.80513	-5602.019593	-5601.661065
^{3}A	-1175.081670	-1174.398961	-5601.468250	-5606.114426	-5605.74582	-5601.976943	-5601.618676

[a]def2-TZVP basis set. [b]m = 4000. [c]With metal 4s and 4p electrons correlated. [d]Without 4s and 4p electrons correlated.

Table S5. Total Energies (in au) of Fe[Cor]Ph

	B3LYP[a]	B3LYP*[a]	DMRG[b]	CASPT2		CCSD(T)	
				3s3p[c]	0s0p[d]	3s3p[c]	0s0p[d]
$^{1}A'$	-2445.643432	-2444.719295	-2446.257865	-2451.483288	-2451.059746	-2446.741881	-2446.312008
$^{3}A'$	-2445.641523	-2444.716674	-2446.252878	-2451.474092	-2451.050983	-2446.736387	-2446.306668
$^{3}A''$	-2445.667068	-2444.743892	-2446.253190	-2451.517409	-2451.091859	-2446.751280	-2446.323670
$^{5}A'$	-2445.634549	-2444.704457	-2446.278095	-2451.477840	-2451.056808	-2446.687927	-2446.265236
$^{5}A''$	-2445.631875	-2444.703677	-2446.229214	-2451.459991	-2451.037292	-2446.691472	-2446.263686
$^{7}A'$	-2445.620958	-2444.687968	-2446.273199	-2451.457866	-2451.038522	-2446.651779	-2446.229849
$^{7}A''$	-2445.617145	-2444.683896	-2446.278590	-2451.453267	-2451.033782	-2446.646868	-2446.224794

[a]def2-TZVP basis set. [b]m = 4000. [c]With metal 3s and 3p electrons correlated. [d]Without 3s and 3p electrons correlated.

Table S6. Total Energies (in au) of Mn[Cor]Ph

	B3LYP[a]	B3LYP*[a]	DMRG[b]	CASPT2		CCSD(T)	
				3s3p[c]	0s0p[d]	3s3p[c]	0s0p[d]
$^2A'$	-2332.928289	-2332.008878	-2332.210547	-2337.409622	-2336.987150	-2332.693465	-2332.273401
$^2A''$	-2332.929706	-2332.009111	-2332.206054	-2337.407817	-2336.985784	-2332.688426	-2332.268996
$^4A'$	-2332.926589	-2332.005679	-2332.211063	-2337.402786	-2336.980588	-2332.689270	-2332.269522
$^4A''$	-2332.968479	-2332.049294	-2332.232638	-2337.477692	-2337.052195	-2332.729169	-2332.312412
$^6A'$	-2332.914322	-2331.990120	-2332.226255	-2337.395916	-2336.974059	-2332.647032	-2332.230561
$^6A''$	-2332.919796	-2331.995913	-2332.221679	-2337.406215	-2336.984970	-2332.651690	-2332.235390

[a]def2-TZVP basis set. [b]*m* = 4000. [c]With metal 3s and 3p electrons correlated. [d]Without 3s and 3p electrons correlated.

Table S7. Total Energies (in au) of $Mo[Cor]Cl_2$

	B3LYP[a]	B3LYP*[a]	DMRG[b]	CASPT2		CCSD(T)	
				4s4p[c]	0s0p[d]	4s4p[c]	0s0p[d]
$^2A'$	-1938.979141	-1938.151773	-5912.311306				
$^2A''$	-1938.935500	-1938.107208	-5912.291058				
$^4A''$	-1938.935320	-1938.104799	-5912.282129				
$^4A'$	-1938.937286	-1938.106544	-5912.294040				

[a]def2-TZVP basis set. [b]*m* = 4000. [c]With metal 4s and 4p electrons correlated. [d]Without 4s and 4p electrons correlated.

Table S8. Relative Energies (kcal/mol) Between Spin States of Mn[Cor]Cl

	B3LYP[a]	B3LYP*[a]	DMRG[b]	CASPT2		CCSD(T)	
				3s3p[c]	0s0p[d]	3s3p[c]	0s0p[d]
$^2A'$	24.2	23.6	29.7	34.4	34.1	18.3	19.7
$^2A''$	23.4	23.3	48.7	37.9	36.5	22.0	19.0
$^4A'$	10.7	12.3	0.7	15.0	14.8	16.3	16.1
$^4A''$	0.0	0.0	0.0	0.0	0.0	0.0	0.0
$^6A'$	10.8	12.5	0.6	14.8	14.5	16.3	16.1
$^6A''$	7.7	9.3	4.1	9.7	8.8	13.5	13.1

[a]def2-TZVP basis set. [b]*m* = 4000. [c]With metal 3s and 3p electrons correlated. [d]Without 3s and 3p electrons correlated.

Table S9. Relative Energies (kcal/mol) Between Spin States of Fe[Cor]Cl

	B3LYP[a]	B3LYP*[a]	DMRG[b]	CASPT2		CCSD(T)	
				3s3p[c]	0s0p[d]	3s3p	0s0p
$^1A'$	18.7	18.5	13.4	20.9	20.8	10.8	10.7
$^3A'$	8.3	9.4	2.4	14.2	13.7	12.0	11.9
$^3A''$	0.0	0.0	0.0	0.0	0.0	0.0	0.0
$^5A'$	10.1	13.4	-22.9	3.1	0.8	27.5	23.9
$^5A''$	6.6	7.7	8.2	12.1	11.2	11.4	11.2
$^7A'$	15.0	18.8	-17.6	11.9	8.4	34.8	31.0
$^7A''$	16.2	20.0	-22.2	14.2	11.6	37.4	33.7

[a]def2-TZVP basis set. [b]*m* = 4000. [c]With metal 3s and 3p electrons correlated. [d]Without 3s and 3p electrons correlated.

Table S10. Relative Energies (kcal/mol) Between Spin States of Fe[Cor]NO

	B3LYP[a]	B3LYP*[a]
^1A	0.0	0.0
^3A	5.8	8.2

[a]def2-TZVP basis set.

Table S11. Relative Energies (kcal/mol) Between Spin States of Ru[Cor]NO

	B3LYP[a]	B3LYP*[a]	DMRG[b]	CASPT2		CCSD(T)	
				3s3p[c]	0s0p[d]	3s3p	0s0p
$^1A'$	0.0	0.0	0.0	0.0	0.0	0.0	0.0
^3A	24.8	25.9	14.1	36.1	37.2	26.8	26.6

[a]def2-TZVP basis set. [b]m = 4000. [c]With metal 4s and 4p electrons correlated. [d]Without 4s and 4p electrons correlated.

Table S12. Relative Energies (kcal/mol) Between Spin States of Fe[Cor]Ph.

	B3LYP[a]	B3LYP*[a]	DMRG[b]	CASPT2		CCSD(T)	
				3s3p[c]	0s0p[d]	3s3p	0s0p
$^1A'$	14.8	15.4	-2.9	21.4	20.2	5.9	7.3
$^3A'$	16.0	17.1	0.2	27.2	25.7	9.3	10.7
$^3A''$	0.0	0.0	0.0	0.0	0.0	0.0	0.0
$^5A'$	20.4	24.7	-15.6	24.8	22.0	39.8	36.7
$^5A''$	22.1	25.2	15.0	36.0	34.2	37.5	37.6
$^7A'$	28.9	35.1	-12.6	37.4	33.5	62.4	58.9
$^7A''$	31.3	37.6	-15.9	40.2	36.4	65.5	62.0

[a]def2-TZVP basis set. [b]m = 4000. [c]With metal 3s and 3p electrons correlated. [d]Without 3s and 3p electrons correlated.

Table S13: Relative Energies (kcal/mol) Between Spin States of Mn[Cor]Ph

	B3LYP[a]	B3LYP*[a]	DMRG[b]	CASPT2		CCSD(T)	
				3s3p[c]	0s0p[d]	3s3p	0s0p
$^2A'$	25.2	25.4	13.9	42.7	40.8	22.4	24.5
$^2A''$	24.3	25.2	16.7	43.8	41.7	25.6	27.2
$^4A'$	26.3	27.4	13.5	47.0	44.9	25.0	26.9
$^4A''$	0.0	0.0	0.0	0.0	0.0	0.0	0.0
$^6A'$	34.0	37.1	4.0	51.3	49.0	51.5	51.4
$^6A''$	30.5	33.5	6.9	44.9	42.2	48.6	48.3

[a]def2-TZVP basis set. [b]m = 4000. [c]With metal 3s and 3p electrons correlated. [d]Without 3s and 3p electrons correlated.

Table S14: Relative Energies (kcal/mol) Between Spin States of Mo[Cor]Cl_2

	B3LYP[a]	B3LYP*[a]	DMRG[b]	CASPT2		CCSD(T)	
				3s3p[c]	0s0p[d]	3s3p	0s0p
$^{2}A'$	0.0	0.0	0.0				
$^{2}A''$	27.4	28.0	12.7				
$^{4}A'$	27.5	29.5	18.3				
$^{4}A''$	26.3	28.4	10.8				

[a]def2-TZVP basis set. [b]m = 4000. [c]With metal 3s and 3p electrons correlated. [d]Without 3s and 3p electrons correlated.

Table S15: Root-mean-square deviation (RMSD) (in Å) between B3LYP and B3LYP* geometries.

	RMSD
Fe[Cor]Cl	
$^1A'$	0.006
$^3A'$	0.004
$^3A''$	0.004
$^5A'$	0.004
$^5A''$	0.004
$^7A'$	0.004
$^7A''$	0.004
Mn[Cor]Cl	
$^2A'$	0.004
$^2A''$	0.024
$^4A'$	0.004
$^4A''$	0.007
$^6A'$	0.004
$^6A''$	0.004
Fe[Cor](NO)	
^1A	0.107
^3A	0.018
Ru[Cor](NO)	
$^1A'$	0.004
^3A	0.004
Fe[Cor]Ph	
$^1A'$	0.005
$^3A'$	0.005
$^3A''$	0.005
$^5A'$	0.037
$^5A''$	0.006
$^7A'$	0.006
$^7A''$	0.013
Mn[Cor]Ph	
$^2A'$	0.015
$^2A''$	0.014
$^4A'$	0.041
$^4A''$	0.005
$^6A'$	0.009
$^6A''$	0.012
Mo[Cor]Cl_2	
$^2A'$	0.004
$^2A''$	0.004
$^4A'$	0.004
$^4A''$	0.004

Table S16: Comparison between B3LYP and experimental geometries[1].

	d(metal-N)		*d*(metal-axial ligand)	
	B3LYP	Exp.	B3LYP	Exp.
Fe[Cor]Cl	1.909/1.938		2.229	
Mn[Cor]Cl	1.936/1.952	1.917/1.930	2.261	2.295
Fe[Cor](NO)	1.919/1.928/1.938/1.953	1.900/1.920	1.719	1.641
Ru[Cor](NO)	1.976/2.014	1.967/1.998	1.796	1.715
Fe[Cor]Ph	1.899/1.919		2.046	
Mn[Cor]Ph	1.910/1.919		1.990	
Mo[Cor]Cl_2	2.053/2.081	2.033/2.064	2.363	2.370

Table S17: Active spaces employed in this study

State	Active space
Fe[Cor]Cl	
$^7A'$	CAS(24,26)
$^7A''$	CAS(24,26)
$^5A'$	CAS(24,26)
$^5A''$	CAS(24,25)
$^3A'$	CAS(24,25)
$^3A''$	CAS(24,25)
$^1A'$	CAS(24,25)
Mn[Cor]Cl	
$^6A'$	CAS(23,25)
$^6A''$	CAS(23,25)
$^4A'$	CAS(23,25)
$^4A''$	CAS(23,25)
$^2A'$	CAS(23,25)
$^2A''$	CAS(24,24)
Fe[Cor](NO)	
^3A	CAS(26,27)
^1A	CAS(26,27)
Ru[Cor](NO)	
^3A	CAS(30,26)
$^1A'$	CAS(30,26)
Fe[Cor]Ph	
$^7A'$	CAS(26,27)
$^7A''$	CAS(26,27)
$^5A'$	CAS(26,27)
$^5A''$	CAS(26,26)
$^3A'$	CAS(26,26)
$^3A''$	CAS(26,26)
$^1A'$	CAS(26,26)
Mn[Cor]Ph	
$^6A'$	CAS(25,26)
$^6A''$	CAS(25,26)
$^4A'$	CAS(25,25)
$^4A''$	CAS(25,25)
$^2A'$	CAS(25,25)
$^2A''$	CAS(25,25)

D. Optimized B3LYP Cartesian coordinates (Å)

(i) Mn[Cor]Cl

**^{2}A′**			
Mn	0.4442972	-0.0021542	0.0000000
Cl	2.6375983	-0.0343098	0.0000000
C	-0.1306826	3.6406880	1.8002888
C	-0.1345992	2.8867423	2.9559894
C	-0.0048081	1.4884328	2.5692001
C	0.0024455	2.7194340	0.7010193
C	0.0024455	2.7194340	-0.7010193
C	-0.1306826	3.6406880	-1.8002888
C	-0.1345992	2.8867423	-2.9559894
C	-0.0048081	1.4884328	-2.5692001
C	-0.0157864	0.2923638	-3.3347963
C	-0.0181789	-0.9786908	-2.7718061
C	-0.1159680	-2.2437467	-3.4873810
C	-0.1456427	-3.2411453	-2.5580451
C	-0.0606608	-2.6130774	-1.2445759
C	-0.0779120	-3.2585262	0.0000000
C	-0.0606608	-2.6130774	1.2445759
C	-0.1456427	-3.2411453	2.5580451
C	-0.1159680	-2.2437467	3.4873810
C	-0.0181789	-0.9786908	2.7718061
C	-0.0157864	0.2923638	3.3347963
N	0.0900617	1.4394557	1.2294451
N	0.0900617	1.4394557	-1.2294451
N	0.0198891	-1.2591504	-1.4100354
N	0.0198891	-1.2591504	1.4100354
H	-0.2284051	4.7217181	1.7221979
H	-0.2261222	3.2534379	3.9772595
H	-0.2284051	4.7217181	-1.7221979
H	-0.2261222	3.2534379	-3.9772595
H	-0.0688286	0.3678229	-4.4219744
H	-0.1711037	-2.3395223	-4.5707562
H	-0.2228817	-4.3134286	-2.7310692
H	-0.1414402	-4.3476779	0.0000000
H	-0.2228817	-4.3134286	2.7310692
H	-0.1711037	-2.3395223	4.5707562
H	-0.0688286	0.3678229	4.4219744
**^{2}A″**			
Mn	0.3919291	0.0045536	0.0000000
Cl	2.5925749	-0.0551072	0.0000000
C	-0.1139362	3.6355319	1.8087854
C	-0.1161490	2.8847980	2.9789817
C	-0.0173897	1.4960348	2.6018761
C	-0.0054953	2.7060061	0.7255170
C	-0.0054953	2.7060061	-0.7255170
C	-0.1139362	3.6355319	-1.8087854
C	-0.1161490	2.8847980	-2.9789817
C	-0.0173897	1.4960348	-2.6018761
C	-0.0383355	0.3122254	-3.3401262
C	-0.0379508	-0.9914747	-2.7747303

C	-0.1239980	-2.2364676	-3.4893409
C	-0.1276180	-3.2488942	-2.5521452
C	-0.0419100	-2.6253113	-1.2594728
C	-0.0544623	-3.2539044	0.0000000
C	-0.0419100	-2.6253113	1.2594728
C	-0.1276180	-3.2488942	2.5521452
C	-0.1239980	-2.2364676	3.4893409
C	-0.0379508	-0.9914747	2.7747303
C	-0.0383355	0.3122254	3.3401262
N	0.0565001	1.4531002	1.2336969
N	0.0565001	1.4531002	-1.2336969
N	0.0144799	-1.2628696	-1.4296567
N	0.0144799	-1.2628696	1.4296567
H	-0.1938499	4.7174537	1.7246243
H	-0.2005664	3.2618363	3.9971081
H	-0.1938499	4.7174537	-1.7246243
H	-0.2005664	3.2618363	-3.9971081
H	-0.0890170	0.3776189	-4.4289865
H	-0.1850395	-2.3339465	-4.5721341
H	-0.1888941	-4.3210632	-2.7312628
H	-0.1117029	-4.3446985	0.0000000
H	-0.1888941	-4.3210632	2.7312628
H	-0.1850395	-2.3339465	4.5721341
H	-0.0890170	0.3776189	4.4289865

^{4}A′

Mn	0.5121543	-0.0048584	0.0000000
Cl	2.8191812	-0.1208455	0.0000000
C	-0.1529300	3.6421954	1.7986261
C	-0.1514935	2.8881148	2.9552041
C	0.0117010	1.4930457	2.5738637
C	0.0118836	2.7246158	0.7018140
C	0.0118836	2.7246158	-0.7018140
C	-0.1529300	3.6421954	-1.7986261
C	-0.1514935	2.8881148	-2.9552041
C	0.0117010	1.4930457	-2.5738637
C	-0.0092662	0.2954993	-3.3432112
C	-0.0140060	-0.9753705	-2.7818209
C	-0.1354933	-2.2432150	-3.4920810
C	-0.1609230	-3.2380909	-2.5606886
C	-0.0504910	-2.6106648	-1.2471031
C	-0.0687471	-3.2559160	0.0000000
C	-0.0504910	-2.6106648	1.2471031
C	-0.1609230	-3.2380909	2.5606886
C	-0.1354933	-2.2432150	3.4920810
C	-0.0140060	-0.9753705	2.7818209
C	-0.0092662	0.2954993	3.3432112
N	0.1279860	1.4518491	1.2371528
N	0.1279860	1.4518491	-1.2371528
N	0.0437123	-1.2626887	-1.4258895
N	0.0437123	-1.2626887	1.4258895
H	-0.2712854	4.7208170	1.7180263
H	-0.2669957	3.2520089	3.9749093
H	-0.2712854	4.7208170	-1.7180263
H	-0.2669957	3.2520089	-3.9749093
H	-0.0775429	0.3738832	-4.4288195

H	-0.2086101	-2.3398503	-4.5741594
H	-0.2557014	-4.3094663	-2.7295856
H	-0.1436772	-4.3437454	0.0000000
H	-0.2557014	-4.3094663	2.7295856
H	-0.2086101	-2.3398503	4.5741594
H	-0.0775429	0.3738832	4.4288195
**^{4}A″**			
Mn	0.4078230	0.0039379	0.0000000
Cl	2.6670919	-0.0881210	0.0000000
C	-0.1275218	3.6312762	1.8085812
C	-0.1083979	2.8799362	2.9757901
C	-0.0203396	1.4928987	2.6006731
C	-0.0476614	2.7080816	0.7176188
C	-0.0476614	2.7080816	-0.7176188
C	-0.1275218	3.6312762	-1.8085812
C	-0.1083979	2.8799362	-2.9757901
C	-0.0203396	1.4928987	-2.6006731
C	-0.0274810	0.3122230	-3.3427766
C	-0.0365640	-0.9844813	-2.7793156
C	-0.1055766	-2.2348651	-3.4874844
C	-0.1292774	-3.2425133	-2.5497925
C	-0.0718802	-2.6209492	-1.2548222
C	-0.0857881	-3.2492914	0.0000000
C	-0.0718802	-2.6209492	1.2548222
C	-0.1292774	-3.2425133	2.5497925
C	-0.1055766	-2.2348651	3.4874844
C	-0.0365640	-0.9844813	2.7793156
C	-0.0274810	0.3122230	3.3427766
N	0.0178548	1.4494258	1.2273713
N	0.0178548	1.4494258	-1.2273713
N	-0.0203870	-1.2540596	-1.4291545
N	-0.0203870	-1.2540596	1.4291545
H	-0.2011229	4.7138717	1.7272887
H	-0.1651146	3.2546024	3.9964899
H	-0.2011229	4.7138717	-1.7272887
H	-0.1651146	3.2546024	-3.9964899
H	-0.0574261	0.3808418	-4.4317754
H	-0.1434480	-2.3340849	-4.5710518
H	-0.1859387	-4.3153250	-2.7255247
H	-0.1285621	-4.3402835	0.0000000
H	-0.1859387	-4.3153250	2.7255247
H	-0.1434480	-2.3340849	4.5710518
H	-0.0574261	0.3808418	4.4317754
**^{6}A′**			
Mn	0.5094387	-0.0043110	0.0000000
Cl	2.8177431	-0.1162565	0.0000000
C	-0.1530623	3.6412042	1.8006400
C	-0.1481378	2.8876212	2.9584248
C	0.0130841	1.4945229	2.5761960
C	0.0091149	2.7232206	0.7031625
C	0.0091149	2.7232206	-0.7031625
C	-0.1530623	3.6412042	-1.8006400
C	-0.1481378	2.8876212	-2.9584248
C	0.0130841	1.4945229	-2.5761960
C	-0.0098271	0.2958695	-3.3437849

C	-0.0151799	-0.9746249	-2.7813393
C	-0.1369179	-2.2439532	-3.4915613
C	-0.1595101	-3.2387556	-2.5608024
C	-0.0491749	-2.6104728	-1.2470269
C	-0.0687748	-3.2559397	0.0000000
C	-0.0491749	-2.6104728	1.2470269
C	-0.1595101	-3.2387556	2.5608024
C	-0.1369179	-2.2439532	3.4915613
C	-0.0151799	-0.9746249	2.7813393
C	-0.0098271	0.2958695	3.3437849
N	0.1241981	1.4534759	1.2364995
N	0.1241981	1.4534759	-1.2364995
N	0.0444463	-1.2619131	-1.4263550
N	0.0444463	-1.2619131	1.4263550
H	-0.2694060	4.7201362	1.7201762
H	-0.2645895	3.2513714	3.9779996
H	-0.2694060	4.7201362	-1.7201762
H	-0.2645895	3.2513714	-3.9779996
H	-0.0781380	0.3731806	-4.4294798
H	-0.2095645	-2.3406713	-4.5737055
H	-0.2548862	-4.3101059	-2.7291197
H	-0.1433056	-4.3437045	0.0000000
H	-0.2548862	-4.3101059	2.7291197
H	-0.2095645	-2.3406713	4.5737055
H	-0.0781380	0.3731806	4.4294798
**^{6}A″**			
Mn	0.4514417	0.0034278	0.0000000
Cl	2.7605650	-0.1168284	0.0000000
C	-0.1464160	3.6370362	1.8092697
C	-0.1270950	2.8853953	2.9788105
C	0.0084369	1.4993927	2.6038465
C	-0.0141327	2.7119153	0.7257907
C	-0.0141327	2.7119153	-0.7257907
C	-0.1464160	3.6370362	-1.8092697
C	-0.1270950	2.8853953	-2.9788105
C	0.0084369	1.4993927	-2.6038465
C	-0.0166070	0.3146114	-3.3439017
C	-0.0223742	-0.9909783	-2.7830852
C	-0.1313593	-2.2360562	-3.4947290
C	-0.1477521	-3.2464299	-2.5552074
C	-0.0484166	-2.6240726	-1.2620957
C	-0.0644812	-3.2527825	0.0000000
C	-0.0484166	-2.6240726	1.2620957
C	-0.1477521	-3.2464299	2.5552074
C	-0.1313593	-2.2360562	3.4947290
C	-0.0223742	-0.9909783	2.7830852
C	-0.0166070	0.3146114	3.3439017
N	0.0883168	1.4636381	1.2372641
N	0.0883168	1.4636381	-1.2372641
N	0.0303521	-1.2675834	-1.4416747
N	0.0303521	-1.2675834	1.4416747
H	-0.2504377	4.7166846	1.7247343
H	-0.2206373	3.2588209	3.9973835
H	-0.2504377	4.7166846	-1.7247343
H	-0.2206373	3.2588209	-3.9973835

H	-0.0807963	0.3836434	-4.4315581
H	-0.1989504	-2.3340937	-4.5769283
H	-0.2311270	-4.3174750	-2.7315767
H	-0.1295341	-4.3427144	0.0000000
H	-0.2311270	-4.3174750	2.7315767
H	-0.1989504	-2.3340937	4.5769283
H	-0.0807963	0.3836434	4.4315581

(ii) Fe[Cor]Cl

**^{1}A′**			
Fe	0.3003523	0.0072094	0.0000000
Cl	2.5176641	0.0947854	0.0000000
C	-0.1182066	3.6407205	1.8067116
C	-0.1017477	2.8831884	2.9578698
C	-0.0177240	1.4828795	2.5629514
C	-0.0451135	2.7188000	0.6997768
C	-0.0451135	2.7188000	-0.6997768
C	-0.1182066	3.6407205	-1.8067116
C	-0.1017477	2.8831884	-2.9578698
C	-0.0177240	1.4828795	-2.5629514
C	-0.0063373	0.2872660	-3.3265199
C	-0.0206578	-0.9817790	-2.7611069
C	-0.0889734	-2.2463860	-3.4815591
C	-0.1323632	-3.2458268	-2.5564891
C	-0.0851761	-2.6198482	-1.2400811
C	-0.1053343	-3.2706745	0.0000000
C	-0.0851761	-2.6198482	1.2400811
C	-0.1323632	-3.2458268	2.5564891
C	-0.0889734	-2.2463860	3.4815591
C	-0.0206578	-0.9817790	2.7611069
C	-0.0063373	0.2872660	3.3265199
N	0.0163823	1.4360892	1.2200131
N	0.0163823	1.4360892	-1.2200131
N	-0.0195139	-1.2646606	-1.3992312
N	-0.0195139	-1.2646606	1.3992312
H	-0.1846362	4.7243394	1.7335767
H	-0.1470434	3.2463609	3.9833898
H	-0.1846362	4.7243394	-1.7335767
H	-0.1470434	3.2463609	-3.9833898
H	-0.0219043	0.3604706	-4.4148957
H	-0.1125912	-2.3385144	-4.5663565
H	-0.1943978	-4.3185902	-2.7319336
H	-0.1526738	-4.3603389	0.0000000
H	-0.1943978	-4.3185902	2.7319336
H	-0.1125912	-2.3385144	4.5663565
H	-0.0219043	0.3604706	4.4148957
**^{3}A′**			
Fe	0.4420552	0.0101273	0.0000000
Cl	2.7016948	-0.0869513	0.0000000
C	-0.1363847	3.6351889	1.7969371
C	-0.1322810	2.8809051	2.9526659
C	-0.0087699	1.4842883	2.5665272
C	-0.0136030	2.7131945	0.6996281

C	-0.0136030	2.7131945	-0.6996281
C	-0.1363847	3.6351889	-1.7969371
C	-0.1322810	2.8809051	-2.9526659
C	-0.0087699	1.4842883	-2.5665272
C	-0.0173463	0.2925412	-3.3364821
C	-0.0240006	-0.9712830	-2.7639700
C	-0.1167768	-2.2372571	-3.4800861
C	-0.1442458	-3.2329000	-2.5513669
C	-0.0635894	-2.6024202	-1.2386838
C	-0.0793726	-3.2551046	0.0000000
C	-0.0635894	-2.6024202	1.2386838
C	-0.1442458	-3.2329000	2.5513669
C	-0.1167768	-2.2372571	3.4800861
C	-0.0240006	-0.9712830	2.7639700
C	-0.0173463	0.2925412	3.3364821
N	0.0746123	1.4344095	1.2268167
N	0.0746123	1.4344095	-1.2268167
N	0.0118954	-1.2504643	-1.4041284
N	0.0118954	-1.2504643	1.4041284
H	-0.2279079	4.7165163	1.7172992
H	-0.2165897	3.2454799	3.9751838
H	-0.2279079	4.7165163	-1.7172992
H	-0.2165897	3.2454799	-3.9751838
H	-0.0631229	0.3661074	-4.4235332
H	-0.1672517	-2.3304109	-4.5638028
H	-0.2178863	-4.3059788	-2.7200594
H	-0.1378807	-4.3439045	0.0000000
H	-0.2178863	-4.3059788	2.7200594
H	-0.1672517	-2.3304109	4.5638028
H	-0.0631229	0.3661074	4.4235332
**^{3}A″**			
Fe	0.3722769	0.0185200	0.0000000
Cl	2.6001959	-0.0532266	0.0000000
C	-0.1219295	3.6240359	1.8051738
C	-0.0992296	2.8735455	2.9720005
C	-0.0309510	1.4863844	2.5938600
C	-0.0632526	2.6962351	0.7170766
C	-0.0632526	2.6962351	-0.7170766
C	-0.1219295	3.6240359	-1.8051738
C	-0.0992296	2.8735455	-2.9720005
C	-0.0309510	1.4863844	-2.5938600
C	-0.0282008	0.3098237	-3.3349342
C	-0.0379949	-0.9826834	-2.7610484
C	-0.0961312	-2.2283696	-3.4765173
C	-0.1232489	-3.2378490	-2.5415262
C	-0.0784823	-2.6138972	-1.2484236
C	-0.0921042	-3.2489059	0.0000000
C	-0.0784823	-2.6138972	1.2484236
C	-0.1232489	-3.2378490	2.5415262
C	-0.0961312	-2.2283696	3.4765173
C	-0.0379949	-0.9826834	2.7610484
C	-0.0282008	0.3098237	3.3349342
N	-0.0086412	1.4367098	1.2202426
N	-0.0086412	1.4367098	-1.2202426
N	-0.0300170	-1.2468878	-1.4109038

N	-0.0300170	-1.2468878	1.4109038
H	-0.1816688	4.7072815	1.7217901
H	-0.1371170	3.2477884	3.9937361
H	-0.1816688	4.7072815	-1.7217901
H	-0.1371170	3.2477884	-3.9937361
H	-0.0445957	0.3731906	-4.4245733
H	-0.1229747	-2.3222478	-4.5609013
H	-0.1707996	-4.3111773	-2.7169825
H	-0.1298990	-4.3401533	0.0000000
H	-0.1707996	-4.3111773	2.7169825
H	-0.1229747	-2.3222478	4.5609013
H	-0.0445957	0.3731906	4.4245733
**^{5}A′**			
Fe	0.8013275	-0.0273649	0.0000000
Cl	3.0021141	-0.1860364	0.0000000
C	-0.1866014	3.6242992	1.7989865
C	-0.1724874	2.8856635	2.9722037
C	0.0444702	1.5045844	2.6065023
C	0.0355487	2.6957330	0.7247457
C	0.0355487	2.6957330	-0.7247457
C	-0.1866014	3.6242992	-1.7989865
C	-0.1724874	2.8856635	-2.9722037
C	0.0444702	1.5045844	-2.6065023
C	-0.0065787	0.3222506	-3.3416288
C	0.0005836	-0.9856267	-2.7800687
C	-0.1637227	-2.2277005	-3.4902392
C	-0.1851805	-3.2368878	-2.5515227
C	-0.0351805	-2.6157332	-1.2620810
C	-0.0691201	-3.2381485	0.0000000
C	-0.0351805	-2.6157332	1.2620810
C	-0.1851805	-3.2368878	2.5515227
C	-0.1637227	-2.2277005	3.4902392
C	0.0005836	-0.9856267	2.7800687
C	-0.0065787	0.3222506	3.3416288
N	0.1934403	1.4623899	1.2413587
N	0.1934403	1.4623899	-1.2413587
N	0.0870198	-1.2566512	-1.4403003
N	0.0870198	-1.2566512	1.4403003
H	-0.3531368	4.6952795	1.7001106
H	-0.3292886	3.2585303	3.9833450
H	-0.3531368	4.6952795	-1.7001106
H	-0.3292886	3.2585303	-3.9833450
H	-0.1274414	0.3895459	-4.4250062
H	-0.2692336	-2.3228760	-4.5698367
H	-0.3102564	-4.3044623	-2.7250565
H	-0.1782306	-4.3251275	0.0000000
H	-0.3102564	-4.3044623	2.7250565
H	-0.2692336	-2.3228760	4.5698367
H	-0.1274414	0.3895459	4.4250062
**^{5}A″**			
Fe	0.3781424	0.0187797	0.0000000
Cl	2.6408305	-0.0564751	0.0000000
C	-0.1314899	3.6288226	1.8045551
C	-0.1064189	2.8770453	2.9744115
C	-0.0133691	1.4902330	2.5963875

C	-0.0453010	2.6996321	0.7230908
C	-0.0453010	2.6996321	-0.7230908
C	-0.1314899	3.6288226	-1.8045551
C	-0.1064189	2.8770453	-2.9744115
C	-0.0133691	1.4902330	-2.5963875
C	-0.0200748	0.3096600	-3.3380883
C	-0.0297432	-0.9871380	-2.7654071
C	-0.1130033	-2.2294736	-3.4823574
C	-0.1341805	-3.2410039	-2.5454435
C	-0.0623147	-2.6168109	-1.2531376
C	-0.0763710	-3.2538241	0.0000000
C	-0.0623147	-2.6168109	1.2531376
C	-0.1341805	-3.2410039	2.5454435
C	-0.1130033	-2.2294736	3.4823574
C	-0.0297432	-0.9871380	2.7654071
C	-0.0200748	0.3096600	3.3380883
N	0.0305975	1.4453969	1.2289447
N	0.0305975	1.4453969	-1.2289447
N	0.0025834	-1.2576565	-1.4196699
N	0.0025834	-1.2576565	1.4196699
H	-0.2068922	4.7107829	1.7199237
H	-0.1640143	3.2519787	3.9950748
H	-0.2068922	4.7107829	-1.7199237
H	-0.1640143	3.2519787	-3.9950748
H	-0.0547026	0.3732643	-4.4272615
H	-0.1597404	-2.3235363	-4.5660077
H	-0.1991915	-4.3132733	-2.7219935
H	-0.1280907	-4.3443271	0.0000000
H	-0.1991915	-4.3132733	2.7219935
H	-0.1597404	-2.3235363	4.5660077
H	-0.0547026	0.3732643	4.4272615

^{7}A′

Fe	0.8305168	-0.0271011	0.0000000
Cl	3.0369834	-0.1982552	0.0000000
C	-0.1998681	3.6220147	1.7911287
C	-0.1871245	2.8823300	2.9675066
C	0.0691946	1.5073392	2.6071460
C	0.0650446	2.6996523	0.7269254
C	0.0650446	2.6996523	-0.7269254
C	-0.1998681	3.6220147	-1.7911287
C	-0.1871245	2.8823300	-2.9675066
C	0.0691946	1.5073392	-2.6071460
C	0.0078848	0.3219491	-3.3446280
C	0.0145830	-0.9856497	-2.7841917
C	-0.1807682	-2.2229925	-3.4912584
C	-0.2009590	-3.2336858	-2.5510197
C	-0.0202010	-2.6158924	-1.2641652
C	-0.0540108	-3.2418359	0.0000000
C	-0.0202010	-2.6158924	1.2641652
C	-0.2009590	-3.2336858	2.5510197
C	-0.1807682	-2.2229925	3.4912584
C	0.0145830	-0.9856497	2.7841917
C	0.0078848	0.3219491	3.3446280
N	0.2499922	1.4707508	1.2506263
N	0.2499922	1.4707508	-1.2506263

N	0.1232008	-1.2638971	-1.4460541
N	0.1232008	-1.2638971	1.4460541
H	-0.3923419	4.6881426	1.6881171
H	-0.3747481	3.2514812	3.9748915
H	-0.3923419	4.6881426	-1.6881171
H	-0.3747481	3.2514812	-3.9748915
H	-0.1348613	0.3906519	-4.4251471
H	-0.3084090	-2.3164403	-4.5686298
H	-0.3488713	-4.2986264	-2.7232568
H	-0.1769844	-4.3270633	0.0000000
H	-0.3488713	-4.2986264	2.7232568
H	-0.3084090	-2.3164403	4.5686298
H	-0.1348613	0.3906519	4.4251471

^{7}A″

Fe	0.8836920	-0.0391939	0.0000000
Cl	3.0920519	-0.2325292	0.0000000
C	-0.2040887	3.6273008	1.7797342
C	-0.2129611	2.8832584	2.9417503
C	0.0655911	1.5021198	2.5738065
C	0.0876720	2.7122196	0.7033831
C	0.0876720	2.7122196	-0.7033831
C	-0.2040887	3.6273008	-1.7797342
C	-0.2129611	2.8832584	-2.9417503
C	0.0655911	1.5021198	-2.5738065
C	0.0125280	0.3041206	-3.3406797
C	0.0148572	-0.9681747	-2.7799197
C	-0.1801224	-2.2304779	-3.4881666
C	-0.2080529	-3.2246818	-2.5567168
C	-0.0302148	-2.5981067	-1.2491677
C	-0.0671505	-3.2398072	0.0000000
C	-0.0302148	-2.5981067	1.2491677
C	-0.2080529	-3.2246818	2.5567168
C	-0.1801224	-2.2304779	3.4881666
C	0.0148572	-0.9681747	2.7799197
C	0.0125280	0.3041206	3.3406797
N	0.2802296	1.4611890	1.2472783
N	0.2802296	1.4611890	-1.2472783
N	0.1224550	-1.2533678	-1.4281893
N	0.1224550	-1.2533678	1.4281893
H	-0.4029633	4.6926977	1.6795824
H	-0.4229293	3.2402912	3.9489555
H	-0.4029633	4.6926977	-1.6795824
H	-0.4229293	3.2402912	-3.9489555
H	-0.1200452	0.3825534	-4.4208147
H	-0.3011454	-2.3229720	-4.5665250
H	-0.3585035	-4.2905578	-2.7206177
H	-0.1932060	-4.3232934	0.0000000
H	-0.3585035	-4.2905578	2.7206177
H	-0.3011454	-2.3229720	4.5665250
H	-0.1200452	0.3825534	4.4208147

(iii) Fe[Cor]NO

**^1A**			
Fe	-0.0056765	0.0090229	-0.0214370
N	0.0431917	0.1380906	1.6922895
O	0.2353184	0.6257163	2.7337846
N	-1.4164606	1.2218417	-0.4900405
N	-1.4172767	-1.2214521	-0.4800480
N	1.2729795	1.4130695	-0.4078712
N	1.2757085	-1.4114493	-0.4154047
C	3.2763343	0.0002837	-0.4250529
C	-2.6660927	0.7167311	-0.6388357
C	-2.6657984	-0.7188143	-0.6335024
C	-3.5867565	1.8043882	-0.7971662
C	-3.5870080	-1.8075692	-0.7890564
C	-2.8410116	2.9717147	-0.7406554
C	-2.8401261	-2.9735018	-0.7300705
C	-1.4623173	2.5944585	-0.5548859
C	-1.4613625	-2.5932763	-0.5454031
C	-0.2848688	3.3343926	-0.5122910
C	-0.2827559	-3.3316403	-0.5085277
C	1.0079847	2.7626785	-0.4585390
C	1.0105533	-2.7591109	-0.4613719
C	2.2562130	3.4773274	-0.5091355
C	2.2587527	-3.4767969	-0.5084558
C	3.2660486	2.5433523	-0.4924177
C	3.2688584	-2.5440009	-0.4927849
C	2.6415274	1.2497324	-0.4293283
C	2.6426151	-1.2497145	-0.4342428
H	4.3682655	0.0014981	-0.4439879
H	-0.3460412	4.4231098	-0.5670501
H	-0.3433382	-4.4204987	-0.5629730
H	-4.6607672	1.7176828	-0.9497323
H	-4.6610289	-1.7220056	-0.9422700
H	-3.2086399	3.9917032	-0.8407075
H	-3.2065639	-3.9941951	-0.8275577
H	2.3503501	4.5604818	-0.5690303
H	2.3517275	-4.5603182	-0.5641684
H	4.3398505	2.7182895	-0.5321001
H	4.3427626	-2.7192134	-0.5288162

(iv) Ru[Cor]NO

**$^1A'$**			
Ru	0.3379930	0.0124020	0.0000000
N	2.0398920	0.1059470	0.0000000
N	-0.1376030	-1.4605850	1.2382120
N	-0.2251790	1.2967110	1.4302740
N	-0.2251790	1.2967110	-1.4302740
N	-0.1376030	-1.4605850	-1.2382120
C	-0.2751530	-2.7231630	0.7132360
C	-0.4705600	-3.6176400	1.8107540
C	-0.4595860	-2.8584610	2.9768860
C	-0.2672420	-1.4829000	2.6074500
C	-0.3002400	-0.2883340	3.3393520

C	-0.3008050	1.0046360	2.7846440
C	-0.4731830	2.2517630	3.4847530
C	-0.5120330	3.2628990	2.5523850
C	-0.3624380	2.6622870	1.2533180
C	-0.3988420	3.2883120	0.0000000
C	-0.3624380	2.6622870	-1.2533180
C	-0.5120330	3.2628990	-2.5523850
C	-0.4731830	2.2517630	-3.4847530
C	-0.3008050	1.0046360	-2.7846440
C	-0.3002400	-0.2883340	-3.3393520
C	-0.2672420	-1.4829000	-2.6074500
C	-0.4595860	-2.8584610	-2.9768860
C	-0.4705600	-3.6176400	-1.8107540
C	-0.2751530	-2.7231630	-0.7132360
H	-0.6297060	-4.6919360	-1.7374600
H	-0.5812110	2.3445920	-4.5644800
H	-0.6506170	4.3274210	-2.7341280
H	-0.6048810	-3.2240040	-3.9921680
H	-0.5812110	2.3445920	4.5644800
H	-0.6297060	-4.6919360	1.7374600
H	-0.6048810	-3.2240040	3.9921680
H	-0.6506170	4.3274210	2.7341280
H	-0.4011930	-0.3558830	4.4243220
H	-0.5166630	4.3737690	0.0000000
H	-0.4011930	-0.3558830	-4.4243220
O	3.2034770	0.1962310	0.0000000

^{3}A

Ru	-0.3125989	0.0069381	0.0011559
N	-2.1097242	-0.1400998	-0.0560072
N	-0.0172122	1.5259100	1.2572599
N	0.1530898	-1.2993906	1.4555448
N	0.2127524	-1.2863405	-1.4441983
N	-0.0496734	1.5127386	-1.2519870
C	0.1079613	2.7819963	0.7248941
C	0.3554625	3.6810525	1.8075534
C	0.3974423	2.9169677	2.9745012
C	0.1842711	1.5437772	2.6033397
C	0.2795408	0.3246833	3.3281178
C	0.2928137	-0.9772488	2.8002486
C	0.5853685	-2.2064312	3.5044468
C	0.6566651	-3.2166094	2.5751843
C	0.4057267	-2.6289122	1.2763422
C	0.4797987	-3.2503756	0.0034782
C	0.4239183	-2.6325039	-1.2567691
C	0.6452891	-3.2236318	-2.5572495
C	0.5803781	-2.2103761	-3.4878563
C	0.3179993	-0.9797803	-2.7772709
C	0.2785466	0.3349487	-3.3267572
C	0.1633865	1.5239671	-2.6001401
C	0.3746614	2.9175129	-2.9679604
C	0.3393600	3.6745416	-1.8083454
C	0.0947277	2.7692519	-0.7181035
H	0.4967078	4.7480179	-1.7254416
H	0.7222817	-2.2923889	-4.5644142
H	0.8457861	-4.2780564	-2.7417219

H	0.5564752	3.2830777	-3.9777812
H	0.7511920	-2.2846940	4.5780055
H	0.5075366	4.7560028	1.7302929
H	0.5891274	3.2816306	3.9824071
H	0.8856718	-4.2658643	2.7550757
H	0.4460105	0.4028890	4.4054015
H	0.6763262	-4.3248826	0.0053880
H	0.4365402	0.4108591	-4.4045226
O	-2.9873345	0.1131069	-0.8021423

(v) Fe[Cor]Ph

$^{1}A'$			
Fe	-0.6206027	-0.0011880	0.0000000
C	-1.0236724	3.6482735	1.8181183
C	-1.0246118	2.8820599	2.9637549
C	-0.9135456	1.4809239	2.5654186
C	-0.9125957	2.7407308	0.7016117
C	-0.9125957	2.7407308	-0.7016117
C	-1.0236724	3.6482735	-1.8181183
C	-1.0246118	2.8820599	-2.9637549
C	-0.9135456	1.4809239	-2.5654186
C	-0.9133966	0.2748533	-3.3234091
C	-0.8998855	-1.0061656	-2.7689019
C	-0.9535726	-2.2720148	-3.4909705
C	-0.9585345	-3.2768394	-2.5681012
C	-0.9046993	-2.6573637	-1.2478904
C	-0.9066535	-3.3038484	0.0000000
C	-0.9046993	-2.6573637	1.2478904
C	-0.9585345	-3.2768394	2.5681012
C	-0.9535726	-2.2720148	3.4909705
C	-0.8998855	-1.0061656	2.7689019
C	-0.9133966	0.2748533	3.3234091
N	-0.8355154	1.4462292	1.2231072
N	-0.8355154	1.4462292	-1.2231072
N	-0.8660997	-1.2988696	-1.4090132
N	-0.8660997	-1.2988696	1.4090132
H	-1.1039236	4.7317102	1.7555957
H	-1.1025735	3.2411315	3.9889713
H	-1.1039236	4.7317102	-1.7555957
H	-1.1025735	3.2411315	-3.9889713
H	-0.9578898	0.3496416	-4.4112665
H	-0.9948188	-2.3647185	-4.5753289
H	-1.0021131	-4.3493987	-2.7510699
H	-0.9344772	-4.3948101	0.0000000
H	-1.0021131	-4.3493987	2.7510699
H	-0.9948188	-2.3647185	4.5753289
H	-0.9578898	0.3496416	4.4112665
C	1.3784875	0.0729404	0.0000000
C	2.0937884	0.0942543	-1.2077841
H	1.5689347	0.0814648	-2.1646393
C	3.4929310	0.1323878	-1.2048642
H	4.0311646	0.1480627	-2.1570580
C	4.1994632	0.1527034	0.0000000

H	5.2920035	0.1849661	0.0000000
C	3.4929310	0.1323878	1.2048642
H	4.0311646	0.1480627	2.1570580
C	2.0937884	0.0942543	1.2077841
H	1.5689347	0.0814648	2.1646393
**^{3}A′**			
Fe	-0.6124301	0.0011374	0.0000000
C	-1.0361411	3.6486169	1.8208301
C	-1.0284222	2.8836033	2.9688145
C	-0.9101440	1.4858645	2.5685370
C	-0.9215588	2.7407284	0.7042312
C	-0.9215588	2.7407284	-0.7042312
C	-1.0361411	3.6486169	-1.8208301
C	-1.0284222	2.8836033	-2.9688145
C	-0.9101440	1.4858645	-2.5685370
C	-0.9076632	0.2783249	-3.3243187
C	-0.8983892	-1.0023433	-2.7672835
C	-0.9547984	-2.2718115	-3.4897807
C	-0.9567637	-3.2763519	-2.5678794
C	-0.9006819	-2.6544614	-1.2484827
C	-0.9025515	-3.3004623	0.0000000
C	-0.9006819	-2.6544614	1.2484827
C	-0.9567637	-3.2763519	2.5678794
C	-0.9547984	-2.2718115	3.4897807
C	-0.8983892	-1.0023433	2.7672835
C	-0.9076632	0.2783249	3.3243187
N	-0.8364614	1.4538104	1.2236044
N	-0.8364614	1.4538104	-1.2236044
N	-0.8619228	-1.2936351	-1.4116562
N	-0.8619228	-1.2936351	1.4116562
H	-1.1236333	4.7315909	1.7579863
H	-1.1083296	3.2422060	3.9938977
H	-1.1236333	4.7315909	-1.7579863
H	-1.1083296	3.2422060	-3.9938977
H	-0.9516157	0.3517149	-4.4122860
H	-0.9970097	-2.3649523	-4.5741509
H	-1.0017232	-4.3488729	-2.7497300
H	-0.9305909	-4.3911753	0.0000000
H	-1.0017232	-4.3488729	2.7497300
H	-0.9970097	-2.3649523	4.5741509
H	-0.9516157	0.3517149	4.4122860
C	1.3810039	0.0741365	0.0000000
C	2.0972284	0.0924280	-1.2076148
H	1.5728532	0.0840707	-2.1648140
C	3.4965274	0.1224670	-1.2047842
H	4.0348058	0.1363195	-2.1569983
C	4.2033130	0.1377387	0.0000000
H	5.2959056	0.1634604	0.0000000
C	3.4965274	0.1224670	1.2047842
H	4.0348058	0.1363195	2.1569983
C	2.0972284	0.0924280	1.2076148
H	1.5728532	0.0840707	2.1648140
**^{3}A″**			
Fe	-0.6486148	0.0204025	0.0000000
C	-1.0382034	3.6345009	1.8107674

C	-1.0145156	2.8707217	2.9749146
C	-0.9560831	1.4885373	2.5939126
C	-0.9930071	2.7292064	0.7120092
C	-0.9930071	2.7292064	-0.7120092
C	-1.0382034	3.6345009	-1.8107674
C	-1.0145156	2.8707217	-2.9749146
C	-0.9560831	1.4885373	-2.5939126
C	-0.9230352	0.3018336	-3.3378211
C	-0.9094209	-0.9836770	-2.7714929
C	-0.9073541	-2.2360536	-3.4783738
C	-0.9104120	-3.2466528	-2.5453943
C	-0.9095322	-2.6350815	-1.2450641
C	-0.9041510	-3.2735391	0.0000000
C	-0.9095322	-2.6350815	1.2450641
C	-0.9104120	-3.2466528	2.5453943
C	-0.9073541	-2.2360536	3.4783738
C	-0.9094209	-0.9836770	2.7714929
C	-0.9230352	0.3018336	3.3378211
N	-0.9426855	1.4459464	1.2200866
N	-0.9426855	1.4459464	-1.2200866
N	-0.9136280	-1.2584225	-1.4066132
N	-0.9136280	-1.2584225	1.4066132
H	-1.0923454	4.7194115	1.7436391
H	-1.0408598	3.2413111	3.9985248
H	-1.0923454	4.7194115	-1.7436391
H	-1.0408598	3.2413111	-3.9985248
H	-0.9259371	0.3682582	-4.4272866
H	-0.9147149	-2.3337019	-4.5629708
H	-0.9128271	-4.3201602	-2.7272450
H	-0.8981316	-4.3650484	0.0000000
H	-0.9128271	-4.3201602	2.7272450
H	-0.9147149	-2.3337019	4.5629708
H	-0.9259371	0.3682582	4.4272866
C	1.3974369	0.0326823	0.0000000
C	2.0810524	0.0497236	-1.2169965
H	1.5445558	0.0371469	-2.1676391
C	3.4806497	0.0854137	-1.2096651
H	4.0221163	0.0991042	-2.1593488
C	4.1802183	0.1031716	0.0000000
H	5.2725794	0.1310687	0.0000000
C	3.4806497	0.0854137	1.2096651
H	4.0221163	0.0991042	2.1593488
C	2.0810524	0.0497236	1.2169965
H	1.5445558	0.0371469	2.1676391
**^{5}A′**			
Fe	-0.3293872	0.0346314	0.0000000
C	-1.2689902	3.6630660	1.8073687
C	-1.2194991	2.9215822	2.9797608
C	-1.0134769	1.5417856	2.6081152
C	-1.0846021	2.7405498	0.7216044
C	-1.0846021	2.7405498	-0.7216044
C	-1.2689902	3.6630660	-1.8073687
C	-1.2194991	2.9215822	-2.9797608
C	-1.0134769	1.5417856	-2.6081152
C	-0.9856076	0.3546048	-3.3460718

C	-0.9375195	-0.9506324	-2.7895994
C	-0.9799267	-2.2053158	-3.4990134
C	-0.9760525	-3.2130949	-2.5594391
C	-0.9274927	-2.5876771	-1.2628655
C	-0.9354839	-3.2088840	0.0000000
C	-0.9274927	-2.5876771	1.2628655
C	-0.9760525	-3.2130949	2.5594391
C	-0.9799267	-2.2053158	3.4990134
C	-0.9375195	-0.9506324	2.7895994
C	-0.9856076	0.3546048	3.3460718
N	-0.9193071	1.5015991	1.2393249
N	-0.9193071	1.5015991	-1.2393249
N	-0.8995033	-1.2208923	-1.4429035
N	-0.8995033	-1.2208923	1.4429035
H	-1.4336179	4.7355290	1.7195379
H	-1.3368967	3.2966530	3.9955370
H	-1.4336179	4.7355290	-1.7195379
H	-1.3368967	3.2966530	-3.9955370
H	-1.0536272	0.4206667	-4.4341962
H	-1.0234399	-2.3104487	-4.5821458
H	-1.0122718	-4.2866555	-2.7380207
H	-0.9652808	-4.3009164	0.0000000
H	-1.0122718	-4.2866555	2.7380207
H	-1.0234399	-2.3104487	4.5821458
H	-1.0536272	0.4206667	4.4341962
C	1.6774765	-0.0326537	0.0000000
C	2.3814262	-0.0470021	-1.2118434
H	1.8453443	-0.0385273	-2.1659999
C	3.7805108	-0.0726276	-1.2113402
H	4.3261319	-0.0833280	-2.1589863
C	4.4795431	-0.0853325	0.0000000
H	5.5723765	-0.1061333	0.0000000
C	3.7805108	-0.0726276	1.2113402
H	4.3261319	-0.0833280	2.1589863
C	2.3814262	-0.0470021	1.2118434
H	1.8453443	-0.0385273	2.1659999
**^{5}A″**			
Fe	-0.5817392	0.0271808	0.0000000
C	-1.1004043	3.6314295	1.8060588
C	-1.0875020	2.8797889	2.9759444
C	-0.9819539	1.4925061	2.5986992
C	-0.9974934	2.7014799	0.7243268
C	-0.9974934	2.7014799	-0.7243268
C	-1.1004043	3.6314295	-1.8060588
C	-1.0875020	2.8797889	-2.9759444
C	-0.9819539	1.4925061	-2.5986992
C	-0.9872697	0.3090240	-3.3389752
C	-0.9566966	-0.9900544	-2.7696204
C	-1.0041515	-2.2351020	-3.4864069
C	-0.9692827	-3.2469135	-2.5491251
C	-0.9032684	-2.6199360	-1.2570609
C	-0.8851160	-3.2556752	0.0000000
C	-0.9032684	-2.6199360	1.2570609
C	-0.9692827	-3.2469135	2.5491251
C	-1.0041515	-2.2351020	3.4864069

C	-0.9566966	-0.9900544	2.7696204
C	-0.9872697	0.3090240	3.3389752
N	-0.9187210	1.4484480	1.2319258
N	-0.9187210	1.4484480	-1.2319258
N	-0.8913329	-1.2611418	-1.4247216
N	-0.8913329	-1.2611418	1.4247216
H	-1.1791865	4.7131961	1.7211397
H	-1.1598532	3.2553703	3.9955360
H	-1.1791865	4.7131961	-1.7211397
H	-1.1598532	3.2553703	-3.9955360
H	-1.0419653	0.3722984	-4.4275440
H	-1.0630038	-2.3322952	-4.5692880
H	-0.9955967	-4.3208195	-2.7264528
H	-0.8935400	-4.3475485	0.0000000
H	-0.9955967	-4.3208195	2.7264528
H	-1.0630038	-2.3322952	4.5692880
H	-1.0419653	0.3722984	4.4275440
C	1.5193607	0.0257882	0.0000000
C	2.2431574	0.0436140	-1.2025873
H	1.7157137	0.0309017	-2.1614548
C	3.6427599	0.0792470	-1.2079984
H	4.1866458	0.0934043	-2.1576344
C	4.3469006	0.0978971	0.0000000
H	5.4399072	0.1269357	0.0000000
C	3.6427599	0.0792470	1.2079984
H	4.1866458	0.0934043	2.1576344
C	2.2431574	0.0436140	1.2025873
H	1.7157137	0.0309017	2.1614548

^{7}A′

Fe	-0.1823692	0.0491217	0.0000000
C	-1.3686971	3.6552381	1.7909891
C	-1.3313617	2.9176652	2.9677162
C	-1.0101684	1.5545850	2.6094195
C	-1.0540520	2.7441488	0.7280592
C	-1.0540520	2.7441488	-0.7280592
C	-1.3686971	3.6552381	-1.7909891
C	-1.3313617	2.9176652	-2.9677162
C	-1.0101684	1.5545850	-2.6094195
C	-1.0269601	0.3653354	-3.3463933
C	-0.9592189	-0.9436664	-2.7897068
C	-1.0981657	-2.1905590	-3.4961265
C	-1.0655011	-3.2002150	-2.5547094
C	-0.9096205	-2.5724312	-1.2681381
C	-0.9104066	-3.1966626	0.0000000
C	-0.9096205	-2.5724312	1.2681381
C	-1.0655011	-3.2002150	2.5547094
C	-1.0981657	-2.1905590	3.4961265
C	-0.9592189	-0.9436664	2.7897068
C	-1.0269601	0.3653354	3.3463933
N	-0.8165122	1.5267955	1.2548937
N	-0.8165122	1.5267955	-1.2548937
N	-0.8315849	-1.2171988	-1.4534826
N	-0.8315849	-1.2171988	1.4534826
H	-1.6092965	4.7114649	1.6862034
H	-1.5447477	3.2787329	3.9729926

H	-1.6092965	4.7114649	-1.6862034
H	-1.5447477	3.2787329	-3.9729926
H	-1.1815836	0.4290954	-4.4257529
H	-1.2258155	-2.2922873	-4.5728574
H	-1.1637265	-4.2710440	-2.7265144
H	-0.9802887	-4.2867950	0.0000000
H	-1.1637265	-4.2710440	2.7265144
H	-1.2258155	-2.2922873	4.5728574
H	-1.1815836	0.4290954	4.4257529
C	1.8515059	-0.0312567	0.0000000
C	2.5725644	-0.0550769	-1.2067233
H	2.0410681	-0.0391386	-2.1636951
C	3.9708694	-0.1003190	-1.2100955
H	4.5163608	-0.1181562	-2.1580749
C	4.6717911	-0.1224421	0.0000000
H	5.7644752	-0.1575223	0.0000000
C	3.9708694	-0.1003190	1.2100955
H	4.5163608	-0.1181562	2.1580749
C	2.5725644	-0.0550769	1.2067233
H	2.0410681	-0.0391386	2.1636951
**^{7}A″**			
Fe	-0.1472388	0.0576052	0.0000000
C	-1.4145426	3.6681811	1.7794371
C	-1.3869376	2.9251530	2.9425421
C	-1.0319565	1.5606793	2.5763547
C	-1.0694219	2.7694636	0.7043871
C	-1.0694219	2.7694636	-0.7043871
C	-1.4145426	3.6681811	-1.7794371
C	-1.3869376	2.9251530	-2.9425421
C	-1.0319565	1.5606793	-2.5763547
C	-1.0237640	0.3584905	-3.3443208
C	-0.9595175	-0.9143930	-2.7854443
C	-1.0884148	-2.1869793	-3.4930783
C	-1.0623087	-3.1804805	-2.5601089
C	-0.9166403	-2.5438386	-1.2521025
C	-0.9174129	-3.1852339	0.0000000
C	-0.9166403	-2.5438386	1.2521025
C	-1.0623087	-3.1804805	2.5601089
C	-1.0884148	-2.1869793	3.4930783
C	-0.9595175	-0.9143930	2.7854443
C	-1.0237640	0.3584905	3.3443208
N	-0.8099178	1.5322651	1.2518188
N	-0.8099178	1.5322651	-1.2518188
N	-0.8356448	-1.1947393	-1.4357576
N	-0.8356448	-1.1947393	1.4357576
H	-1.6714611	4.7210797	1.6784969
H	-1.6210069	3.2715506	3.9482423
H	-1.6714611	4.7210797	-1.6784969
H	-1.6210069	3.2715506	-3.9482423
H	-1.1618553	0.4312851	-4.4242382
H	-1.2053638	-2.2882250	-4.5711832
H	-1.1564204	-4.2529057	-2.7239887
H	-0.9848710	-4.2739815	0.0000000
H	-1.1564204	-4.2529057	2.7239887
H	-1.2053638	-2.2882250	4.5711832

H	-1.1618553	0.4312851	4.4242382
C	1.8866149	-0.0455148	0.0000000
C	2.6105769	-0.0794980	-1.2056816
H	2.0800738	-0.0576566	-2.1637242
C	4.0082301	-0.1418196	-1.2098872
H	4.5535169	-0.1667843	-2.1579440
C	4.7093301	-0.1725881	0.0000000
H	5.8015380	-0.2215622	0.0000000
C	4.0082301	-0.1418196	1.2098872
H	4.5535169	-0.1667843	2.1579440
C	2.6105769	-0.0794980	1.2056816
H	2.0800738	-0.0576566	2.1637242

(vi) Mn[Cor]Ph

$^{2}A'$			
Mn	-0.5186987	0.0023327	0.0000000
C	-1.1046890	3.6376817	1.7999683
C	-1.1011193	2.8812385	2.9551610
C	-0.9382554	1.4852644	2.5693015
C	-0.9409465	2.7229158	0.7008732
C	-0.9409465	2.7229158	-0.7008732
C	-1.1046890	3.6376817	-1.7999683
C	-1.1011193	2.8812385	-2.9551610
C	-0.9382554	1.4852644	-2.5693015
C	-0.9284869	0.2859944	-3.3342809
C	-0.8973763	-0.9884804	-2.7745261
C	-0.9549911	-2.2546957	-3.4907267
C	-0.9474122	-3.2541055	-2.5608889
C	-0.8823070	-2.6257472	-1.2465569
C	-0.8788753	-3.2706120	0.0000000
C	-0.8823070	-2.6257472	1.2465569
C	-0.9474122	-3.2541055	2.5608889
C	-0.9549911	-2.2546957	3.4907267
C	-0.8973763	-0.9884804	2.7745261
C	-0.9284869	0.2859944	3.3342809
N	-0.8244113	1.4404548	1.2321991
N	-0.8244113	1.4404548	-1.2321991
N	-0.8456297	-1.2706130	-1.4128691
N	-0.8456297	-1.2706130	1.4128691
H	-1.2290633	4.7161483	1.7240527
H	-1.2115425	3.2460135	3.9754344
H	-1.2290633	4.7161483	-1.7240527
H	-1.2115425	3.2460135	-3.9754344
H	-0.9917724	0.3608215	-4.4211046
H	-1.0104472	-2.3532103	-4.5739151
H	-0.9866970	-4.3281348	-2.7369452
H	-0.9076367	-4.3614005	0.0000000
H	-0.9866970	-4.3281348	2.7369452
H	-1.0104472	-2.3532103	4.5739151
H	-0.9917724	0.3608215	4.4211046
C	1.4394199	0.0203582	0.0000000
C	2.1536161	0.0211300	-1.2079885
H	1.6236167	0.0198083	-2.1628560
C	3.5526350	0.0216818	-1.2077849

H	4.0950074	0.0205865	-2.1576422
C	4.2566295	0.0212428	0.0000000
H	5.3497050	0.0185736	0.0000000
C	3.5526350	0.0216818	1.2077849
H	4.0950074	0.0205865	2.1576422
C	2.1536161	0.0211300	1.2079885
H	1.6236167	0.0198083	2.1628560
**$^{2}A''$**			
Mn	-0.5609653	-0.0132739	0.0000000
C	-1.0442816	3.6181070	1.8137693
C	-1.0380352	2.8666173	2.9830488
C	-0.9275280	1.4775188	2.6027124
C	-0.9282182	2.6898582	0.7269443
C	-0.9282182	2.6898582	-0.7269443
C	-1.0442816	3.6181070	-1.8137693
C	-1.0380352	2.8666173	-2.9830488
C	-0.9275280	1.4775188	-2.6027124
C	-0.9434911	0.2885921	-3.3366851
C	-0.9148690	-1.0202766	-2.7753965
C	-0.9763348	-2.2668829	-3.4925827
C	-0.9455270	-3.2811199	-2.5568862
C	-0.8703349	-2.6565639	-1.2625471
C	-0.8622429	-3.2840534	0.0000000
C	-0.8703349	-2.6565639	1.2625471
C	-0.9455270	-3.2811199	2.5568862
C	-0.9763348	-2.2668829	3.4925827
C	-0.9148690	-1.0202766	2.7753965
C	-0.9434911	0.2885921	3.3366851
N	-0.8487206	1.4385620	1.2350278
N	-0.8487206	1.4385620	-1.2350278
N	-0.8448483	-1.2938889	-1.4323289
N	-0.8448483	-1.2938889	1.4323289
H	-1.1315137	4.6997984	1.7323356
H	-1.1208843	3.2434097	4.0014941
H	-1.1315137	4.6997984	-1.7323356
H	-1.1208843	3.2434097	-4.0014941
H	-1.0097917	0.3528033	-4.4250796
H	-1.0441669	-2.3658123	-4.5749885
H	-0.9811114	-4.3540594	-2.7390967
H	-0.8874296	-4.3763148	0.0000000
H	-0.9811114	-4.3540594	2.7390967
H	-1.0441669	-2.3658123	4.5749885
H	-1.0097917	0.3528033	4.4250796
C	1.3985511	0.0206694	0.0000000
C	2.1134441	0.0421227	-1.2073887
H	1.5859254	0.0254924	-2.1622922
C	3.5116562	0.0878290	-1.2072046
H	4.0527347	0.1052125	-2.1575491
C	4.2157194	0.1113066	0.0000000
H	5.3081597	0.1470274	0.0000000
C	3.5116562	0.0878290	1.2072046
H	4.0527347	0.1052125	2.1575491
C	2.1134441	0.0421227	1.2073887
H	1.5859254	0.0254924	2.1622922
**$^{4}A'$**			

Mn	-0.5116741	0.0016151	0.0000000
C	-1.1141824	3.6358218	1.8055080
C	-1.0960173	2.8821267	2.9631510
C	-0.9307330	1.4892691	2.5735046
C	-0.9517045	2.7193913	0.7042254
C	-0.9517045	2.7193913	-0.7042254
C	-1.1141824	3.6358218	-1.8055080
C	-1.0960173	2.8821267	-2.9631510
C	-0.9307330	1.4892691	-2.5735046
C	-0.9245801	0.2863129	-3.3349987
C	-0.9008643	-0.9876332	-2.7724220
C	-0.9658141	-2.2590679	-3.4899504
C	-0.9530169	-3.2578755	-2.5627089
C	-0.8820108	-2.6261752	-1.2473618
C	-0.8800965	-3.2712752	0.0000000
C	-0.8820108	-2.6261752	1.2473618
C	-0.9530169	-3.2578755	2.5627089
C	-0.9658141	-2.2590679	3.4899504
C	-0.9008643	-0.9876332	2.7724220
C	-0.9245801	0.2863129	3.3349987
N	-0.8291246	1.4469356	1.2322135
N	-0.8291246	1.4469356	-1.2322135
N	-0.8423465	-1.2695393	-1.4158388
N	-0.8423465	-1.2695393	1.4158388
H	-1.2412267	4.7141708	1.7294447
H	-1.2053166	3.2464673	3.9834800
H	-1.2412267	4.7141708	-1.7294447
H	-1.2053166	3.2464673	-3.9834800
H	-0.9877057	0.3588362	-4.4219902
H	-1.0241962	-2.3572157	-4.5731386
H	-0.9977909	-4.3317152	-2.7373534
H	-0.9115828	-4.3618172	0.0000000
H	-0.9977909	-4.3317152	2.7373534
H	-1.0241962	-2.3572157	4.5731386
H	-0.9877057	0.3588362	4.4219902
C	1.4472187	0.0229275	0.0000000
C	2.1628008	0.0238721	-1.2073957
H	1.6332204	0.0246261	-2.1626837
C	3.5618386	0.0230794	-1.2076722
H	4.1042587	0.0225706	-2.1575402
C	4.2660415	0.0215240	0.0000000
H	5.3591174	0.0185103	0.0000000
C	3.5618386	0.0230794	1.2076722
H	4.1042587	0.0225706	2.1575402
C	2.1628008	0.0238721	1.2073957
H	1.6332204	0.0246261	2.1626837

^{4}A″

Mn	-0.6436805	-0.0075801	0.0000000
C	-1.0174728	3.6133827	1.8030608
C	-1.0012434	2.8556422	2.9718567
C	-0.9505286	1.4713912	2.5989422
C	-0.9770240	2.7007732	0.7107753
C	-0.9770240	2.7007732	-0.7107753
C	-1.0174728	3.6133827	-1.8030608
C	-1.0012434	2.8556422	-2.9718567

C	-0.9505286	1.4713912	-2.5989422
C	-0.9257676	0.2872839	-3.3492796
C	-0.9124782	-0.9956262	-2.7848406
C	-0.9014368	-2.2525444	-3.4835969
C	-0.8888302	-3.2584375	-2.5455251
C	-0.8885378	-2.6425988	-1.2466250
C	-0.8730429	-3.2774127	0.0000000
C	-0.8885378	-2.6425988	1.2466250
C	-0.8888302	-3.2584375	2.5455251
C	-0.9014368	-2.2525444	3.4835969
C	-0.9124782	-0.9956262	2.7848406
C	-0.9257676	0.2872839	3.3492796
N	-0.9382356	1.4256238	1.2277899
N	-0.9382356	1.4256238	-1.2277899
N	-0.9110686	-1.2691474	-1.4210252
N	-0.9110686	-1.2691474	1.4210252
H	-1.0630999	4.6981633	1.7287114
H	-1.0304942	3.2311473	3.9935506
H	-1.0630999	4.6981633	-1.7287114
H	-1.0304942	3.2311473	-3.9935506
H	-0.9293434	0.3572586	-4.4381428
H	-0.9123513	-2.3560712	-4.5675635
H	-0.8826324	-4.3327471	-2.7221072
H	-0.8537354	-4.3684302	0.0000000
H	-0.8826324	-4.3327471	2.7221072
H	-0.9123513	-2.3560712	4.5675635
H	-0.9293434	0.3572586	4.4381428
C	1.3465430	0.0146791	0.0000000
C	2.0380210	0.0318574	-1.2143713
H	1.5051745	0.0217665	-2.1666351
C	3.4369974	0.0637804	-1.2092567
H	3.9784680	0.0767740	-2.1588717
C	4.1376036	0.0793235	0.0000000
H	5.2300797	0.1040768	0.0000000
C	3.4369974	0.0637804	1.2092567
H	3.9784680	0.0767740	2.1588717
C	2.0380210	0.0318574	1.2143713
H	1.5051745	0.0217665	2.1666351
**^{6}A′**			
Mn	-0.4889899	0.0233038	0.0000000
C	-1.2175026	3.6478862	1.8037471
C	-1.1914949	2.8946910	2.9617297
C	-1.0088275	1.5044572	2.5784161
C	-1.0481641	2.7315420	0.7039733
C	-1.0481641	2.7315420	-0.7039733
C	-1.2175026	3.6478862	-1.8037471
C	-1.1914949	2.8946910	-2.9617297
C	-1.0088275	1.5044572	-2.5784161
C	-0.9966181	0.3033359	-3.3462143
C	-0.9597811	-0.9685248	-2.7859335
C	-1.0260483	-2.2431322	-3.4959450
C	-0.9953400	-3.2372447	-2.5639679
C	-0.9091306	-2.6032927	-1.2501188
C	-0.8919549	-3.2480527	0.0000000
C	-0.9091306	-2.6032927	1.2501188

C	-0.9953400	-3.2372447	2.5639679
C	-1.0260483	-2.2431322	3.4959450
C	-0.9597811	-0.9685248	2.7859335
C	-0.9966181	0.3033359	3.3462143
N	-0.9096766	1.4649269	1.2369097
N	-0.9096766	1.4649269	-1.2369097
N	-0.8813527	-1.2532569	-1.4324162
N	-0.8813527	-1.2532569	1.4324162
H	-1.3508133	4.7249677	1.7246673
H	-1.3055938	3.2575859	3.9818922
H	-1.3508133	4.7249677	-1.7246673
H	-1.3055938	3.2575859	-3.9818922
H	-1.0653621	0.3796423	-4.4320280
H	-1.0973155	-2.3450213	-4.5777246
H	-1.0399230	-4.3119727	-2.7321615
H	-0.9116365	-4.3381841	0.0000000
H	-1.0399230	-4.3119727	2.7321615
H	-1.0973155	-2.3450213	4.5777246
H	-1.0653621	0.3796423	4.4320280
C	1.6327324	-0.0088123	0.0000000
C	2.3720004	-0.0180713	-1.1981732
H	1.8483957	-0.0119987	-2.1617571
C	3.7719017	-0.0357277	-1.2072749
H	4.3160713	-0.0427267	-2.1572287
C	4.4777375	-0.0449582	0.0000000
H	5.5712613	-0.0594274	0.0000000
C	3.7719017	-0.0357277	1.2072749
H	4.3160713	-0.0427267	2.1572287
C	2.3720004	-0.0180713	1.1981732
H	1.8483957	-0.0119987	2.1617571
**^{6}A″**			
Mn	-0.5296280	0.0310829	0.0000000
C	-1.2125858	3.6457077	1.8103449
C	-1.1756167	2.8951669	2.9799401
C	-1.0021469	1.5125602	2.6055949
C	-1.0540185	2.7232709	0.7264055
C	-1.0540185	2.7232709	-0.7264055
C	-1.2125858	3.6457077	-1.8103449
C	-1.1756167	2.8951669	-2.9799401
C	-1.0021469	1.5125602	-2.6055949
C	-0.9946740	0.3259181	-3.3456971
C	-0.9503598	-0.9806012	-2.7885750
C	-1.0034371	-2.2295839	-3.4997973
C	-0.9681707	-3.2393076	-2.5587732
C	-0.8939824	-2.6123176	-1.2658482
C	-0.8764871	-3.2405043	0.0000000
C	-0.8939824	-2.6123176	1.2658482
C	-0.9681707	-3.2393076	2.5587732
C	-1.0034371	-2.2295839	3.4997973
C	-0.9503598	-0.9806012	2.7885750
C	-0.9946740	0.3259181	3.3456971
N	-0.9184433	1.4784508	1.2388399
N	-0.9184433	1.4784508	-1.2388399
N	-0.8794373	-1.2559319	-1.4468371
N	-0.8794373	-1.2559319	1.4468371

H	-1.3441835	4.7223369	1.7256898
H	-1.2804715	3.2668833	3.9980751
H	-1.3441835	4.7223369	-1.7256898
H	-1.2804715	3.2668833	-3.9980751
H	-1.0650675	0.3941867	-4.4331322
H	-1.0680346	-2.3324995	-4.5817403
H	-1.0023398	-4.3130686	-2.7353454
H	-0.8873063	-4.3323157	0.0000000
H	-1.0023398	-4.3130686	2.7353454
H	-1.0680346	-2.3324995	4.5817403
H	-1.0650675	0.3941867	4.4331322
C	1.5983584	-0.0110510	0.0000000
C	2.3375809	-0.0244270	-1.1979679
H	1.8147389	-0.0158363	-2.1615730
C	3.7372768	-0.0501240	-1.2071483
H	4.2810592	-0.0606344	-2.1572369
C	4.4431480	-0.0632844	0.0000000
H	5.5365422	-0.0842267	0.0000000
C	3.7372768	-0.0501240	1.2071483
H	4.2810592	-0.0606344	2.1572369
C	2.3375809	-0.0244270	1.1979679
H	1.8147389	-0.0158363	2.1615730

(vii) Mo[Cor]Cl_2

$^{2}A'$			
Mo	-0.0525124	-0.9661625	0.0000000
Cl	-0.0918615	-2.7503355	1.5482198
Cl	-0.0918615	-2.7503355	-1.5482198
C	-2.7050804	0.1699291	0.7053059
C	-3.5912267	0.4318154	1.7892950
C	-2.8378542	0.3732270	2.9529170
C	-1.4836198	0.0880915	2.5854322
C	-0.3115455	0.0892666	3.3275435
C	0.9652867	0.0285458	2.7545729
C	2.2101154	0.1606359	3.4529764
C	3.2197474	0.1290322	2.5221555
C	2.6085713	-0.0178753	1.2353011
C	3.2494666	-0.0176919	0.0000000
C	2.6085713	-0.0178753	-1.2353011
C	3.2197474	0.1290322	-2.5221555
C	2.2101154	0.1606359	-3.4529764
C	0.9652867	0.0285458	-2.7545729
C	-0.3115455	0.0892666	-3.3275435
C	-1.4836198	0.0880915	-2.5854322
C	-2.8378542	0.3732270	-2.9529170
C	-3.5912267	0.4318154	-1.7892950
C	-2.7050804	0.1699291	-0.7053059
N	1.2263152	-0.0993366	1.3943355
N	1.2263152	-0.0993366	-1.3943355
N	-1.4494795	-0.0705218	-1.2083369
N	-1.4494795	-0.0705218	1.2083369
H	2.2994625	0.2890088	4.5303621
H	2.2994625	0.2890088	-4.5303621

H	4.2908796	0.2229620	2.6905781
H	4.2908796	0.2229620	-2.6905781
H	-3.1836031	0.5547471	-3.9690546
H	-3.1836031	0.5547471	3.9690546
H	-4.6514135	0.6606649	1.7026516
H	-4.6514135	0.6606649	-1.7026516
H	4.3384162	0.0506808	0.0000000
H	-0.3734116	0.2207579	-4.4087388
H	-0.3734116	0.2207579	4.4087388
**$^{2}A''$**			
Mo	-0.0372822	-1.0616038	0.0000000
Cl	-0.0527551	-2.8695669	1.5597522
Cl	-0.0527551	-2.8695669	-1.5597522
C	-2.6999224	0.1460897	0.6968206
C	-3.5890582	0.4727461	1.7791928
C	-2.8420460	0.4178040	2.9353902
C	-1.4848307	0.0676682	2.5626354
C	-0.3054945	0.0551854	3.3353457
C	0.9569269	-0.0016706	2.7671718
C	2.2187653	0.1690226	3.4690986
C	3.2107212	0.1661874	2.5364677
C	2.5783200	-0.0092482	1.2374302
C	3.2275180	0.0032733	0.0000000
C	2.5783200	-0.0092482	-1.2374302
C	3.2107212	0.1661874	-2.5364677
C	2.2187653	0.1690226	-3.4690986
C	0.9569269	-0.0016706	-2.7671718
C	-0.3054945	0.0551854	-3.3353457
C	-1.4848307	0.0676682	-2.5626354
C	-2.8420460	0.4178040	-2.9353902
C	-3.5890582	0.4727461	-1.7791928
C	-2.6999224	0.1460897	-0.6968206
N	1.2220784	-0.1398158	1.4073363
N	1.2220784	-0.1398158	-1.4073363
N	-1.4495214	-0.1368046	-1.2226491
N	-1.4495214	-0.1368046	1.2226491
H	2.3118214	0.2994454	4.5460852
H	2.3118214	0.2994454	-4.5460852
H	4.2809024	0.2906458	2.6919511
H	4.2809024	0.2906458	-2.6919511
H	-3.1780960	0.6301494	-3.9488719
H	-3.1780960	0.6301494	3.9488719
H	-4.6402396	0.7370496	1.6835107
H	-4.6402396	0.7370496	-1.6835107
H	4.3132663	0.1045731	0.0000000
H	-0.3803555	0.1960196	-4.4141995
H	-0.3803555	0.1960196	4.4141995
**$^{4}A'$**			
Mo	-0.0382477	-1.1178506	0.0000000
Cl	-0.1299696	-2.8595867	1.6059666
Cl	-0.1299696	-2.8595867	-1.6059666
C	-2.6897057	0.0953346	0.7205819
C	-3.5848229	0.4343818	1.7769913
C	-2.8336270	0.4063396	2.9457128
C	-1.4845168	0.0617301	2.5869453

C	-0.3066236	0.1117745	3.3267059
C	0.9836858	0.0440485	2.7578008
C	2.2181284	0.2323821	3.4579908
C	3.2323265	0.1647744	2.5244328
C	2.6132356	-0.0536896	1.2501238
C	3.2582052	-0.0778917	0.0000000
C	2.6132356	-0.0536896	-1.2501238
C	3.2323265	0.1647744	-2.5244328
C	2.2181284	0.2323821	-3.4579908
C	0.9836858	0.0440485	-2.7578008
C	-0.3066236	0.1117745	-3.3267059
C	-1.4845168	0.0617301	-2.5869453
C	-2.8336270	0.4063396	-2.9457128
C	-3.5848229	0.4343818	-1.7769913
C	-2.6897057	0.0953346	-0.7205819
N	1.2525821	-0.1433234	1.4179095
N	1.2525821	-0.1433234	-1.4179095
N	-1.4598588	-0.1573425	-1.2298986
N	-1.4598588	-0.1573425	1.2298986
H	2.3061533	0.4157774	4.5274774
H	2.3061533	0.4157774	-4.5274774
H	4.3018785	0.2840775	2.6879871
H	4.3018785	0.2840775	-2.6879871
H	-3.1733047	0.6485430	-3.9515232
H	-3.1733047	0.6485430	3.9515232
H	-4.6360026	0.6924448	1.6704476
H	-4.6360026	0.6924448	-1.6704476
H	4.3472829	-0.0106150	0.0000000
H	-0.3700894	0.2968784	-4.4003646
H	-0.3700894	0.2968784	4.4003646
**^{4}A″**			
Mo	-0.0365642	-1.1355184	0.0000000
Cl	-0.1304335	-2.8813790	1.6112563
Cl	-0.1304335	-2.8813790	-1.6112563
C	-2.6997931	0.1001284	0.6974788
C	-3.5818576	0.4665571	1.7738012
C	-2.8282396	0.4310492	2.9268220
C	-1.4797882	0.0487874	2.5580265
C	-0.2916089	0.0595040	3.3273853
C	0.9681719	0.0053133	2.7610313
C	2.2249920	0.2087512	3.4621669
C	3.2202333	0.1924139	2.5335212
C	2.5972260	-0.0254908	1.2349145
C	3.2561322	-0.0294091	0.0000000
C	2.5972260	-0.0254908	-1.2349145
C	3.2202333	0.1924139	-2.5335212
C	2.2249920	0.2087512	-3.4621669
C	0.9681719	0.0053133	-2.7610313
C	-0.2916089	0.0595040	-3.3273853
C	-1.4797882	0.0487874	-2.5580265
C	-2.8282396	0.4310492	-2.9268220
C	-3.5818576	0.4665571	-1.7738012
C	-2.6997931	0.1001284	-0.6974788
N	1.2477733	-0.1652673	1.4044648
N	1.2477733	-0.1652673	-1.4044648

N	-1.4558084	-0.1879975	-1.2255785
N	-1.4558084	-0.1879975	1.2255785
H	2.3105045	0.3734242	4.5350046
H	2.3105045	0.3734242	-4.5350046
H	4.2876359	0.3398603	2.6879244
H	4.2876359	0.3398603	-2.6879244
H	-3.1524746	0.6807336	-3.9356346
H	-3.1524746	0.6807336	3.9356346
H	-4.6282823	0.7464561	1.6730449
H	-4.6282823	0.7464561	-1.6730449
H	4.3407205	0.0765214	0.0000000
H	-0.3653056	0.2227248	-4.4029951
H	-0.3653056	0.2227248	4.4029951

(1) Alemayehu, A. B.; Thomas, K. E.; Einrem, R. F.; Ghosh, A. The Story of 5d Metallocorroles: From Metal–Ligand Misfits to New Building Blocks for Cancer Phototherapeutics. *Acc. Chem. Res.* **2021**, *54* (15), 3095–3107.
